# Environmental determinants of population health in urban settings. A systematic review

**DOI:** 10.1186/s12889-020-08905-0

**Published:** 2020-06-03

**Authors:** Marta Salgado, Joana Madureira, Ana Sofia Mendes, Anália Torres, João Paulo Teixeira, Mónica Duarte Oliveira

**Affiliations:** 1 Institute of Environmental Health (ISAMB), Faculty of Medicine of the University of Lisbon (FMUL), Universidade de Lisboa, Av. Prof. Egas Moniz, 1649-028 Lisbon, Portugal; 2 Environmental Health Department, National Institute of Health, R. de Alexandre Herculano 321, 4000-055 Porto, Portugal; 3 EPIUnit - Instituto de Saúde Pública, Universidade do Porto, Rua das Taipas, 135, Porto, Portugal; 4 VALORSUL, Estação Mercadorias Bobadela, Plataforma Ribeirinha CP, 2696-801 Lisbon, São João da Talha Portugal; 5 CEG-IST, Centro de Estudos de Gestão do Instituto Superior Técnico, Universidade de Lisboa, Av. Rovisco Pais, 1, 1049-001 Lisbon, Portugal

**Keywords:** Population health, Urban settings, Environmental determinants, Systematic review

## Abstract

**Background:**

Population health is influenced by interactions between environmental determinants, which are captured by dimensions and indicators. This study aims to systematically review key environmental determinants and respective dimensions and indicators, relevant to evaluate population health in urban settings, and to understand their potential implications into policies.

**Methods:**

A search of literature published between 2008 and 2018 was conducted in PubMed, Web of Science, Scopus and SciELO Portugal databases, on studies with evidence on association between an environmental determinant and a health outcome in urban contexts. Health determinants, dimensions and indicators researched in the selected studies were synthetized, and associations analyzed. An independent assessment of quality of the studies was performed. Key conclusions and policy recommendations were extracted to build a framework to analyze environment related population health and policies in urban settings.

**Results:**

Ninety four studies of varied methodological approaches and quality met the inclusion criteria. The review identified positive associations between all environmental determinants -socioeconomic, built environment, natural environment, healthcare, behaviors, and health outcomes - overall mortality and morbidity, in urban settings. Improvements in income, education, air quality, occupation status, mobility and smoking habits indicators have positive impact in overall mortality and chronic diseases morbidity indicators. Initiatives to improve population health in which policymakers can be more evidence-informed include socioeconomic, natural environment and built environment determinants.

**Conclusions:**

There is scope and need to further explore which environmental determinants and dimensions most contribute to population health to create a series of robust evidence-based measures to better inform urban planning policies.

## Background

Assuring the health of the public goes beyond focusing on the health status of individuals; it requires a population health approach. Population health refers to “health outcomes and their distribution in a population. These outcomes are achieved by patterns of health determinants” [1]. Recent studies and socio-ecological models have been demonstrating that population health is influenced by economic factors, employment, education status, access to green spaces, walkability, water and air quality and individual behavior [2–7]. This wide range of factors can be considered as environment because formally, everything other than the genome is or can be connoted as part of the environment [8]. Taking this broad perspective of environment and perceiving it as relevant to population health [9], environmental determinants include the physical, chemical and biological factors external to the individual, as well as all the other factors impacting behaviors in order to prevent diseases and create healthy environments [10]. Thus, including socioeconomic dimensions- education, employment, income, racial segregation, healthcare dimensions- access to hospital care, health insurance, and behavior dimensions- alcohol consumption, nutrition, physical activity, and smoking habits. The complex and dynamic interaction between environmental determinants and health outcomes are known to affect the development of good livelihood, the building of a sustainable workforce and resilient communities [11–14].

The impact of urban settings on population health has been increasing as more people live in cities and towns than in rural areas [15, 16]. As reported by the United Nations [17], in 2018 about half of the world’s population lived in urban areas but, by 2030, the numbers are expected to increase to two-thirds. Hartley (2004) [18] has documented a difference between urban and rural health frequently expressed in terms of determinants as medical care, built environment, natural environment, and socioeconomic status. Urban settings offer a high variety of opportunities, jobs and services, but the diversity, urban segregation and heterogeneous socioeconomic characteristics contribute to inequalities in health [19]. Population health has changed as the cities become bigger leading to changes in population heterogeneity, environment and society with impact on health and have for long been a serious health policy concern in many countries because there is no consensus on what can be routinely done to overcome intra-urban inequalities in health, their distributions within the country and with other countries [20]. Population health equity is also often dependent on political decision-making [21]. The increasing concern about the influence of context on health [16] requests for the integration of population health into urban planning as an essential goal to improve related-policymaking decisions, to foster healthier lifestyles and to avoid major health risks [22, 23].

An integrated and holistic overview is necessary to facilitate a systematic examination of population health and its multiple environmental determinants in urban contexts, so that it is possible to track new evidence [24, 25] and to foster adapted research and policy development into sustainability [26].

Therefore, we conducted a systematic review of literature to identify which key environmental determinants (socioeconomic, built environment, natural environment, health behaviors and healthcare) and respective dimensions and indicators (used to operationalize the measurement of determinants) are associated with human health outcomes, entailing overall mortality and morbidity, in urban settings. The review enables an informed discussion about relevant environmental health determinants, dimensions, and indicators for urban settings and how these factors interrelate and how they may be tackled through policies defined for the urban context.

## Methods

**Fig. 1:**
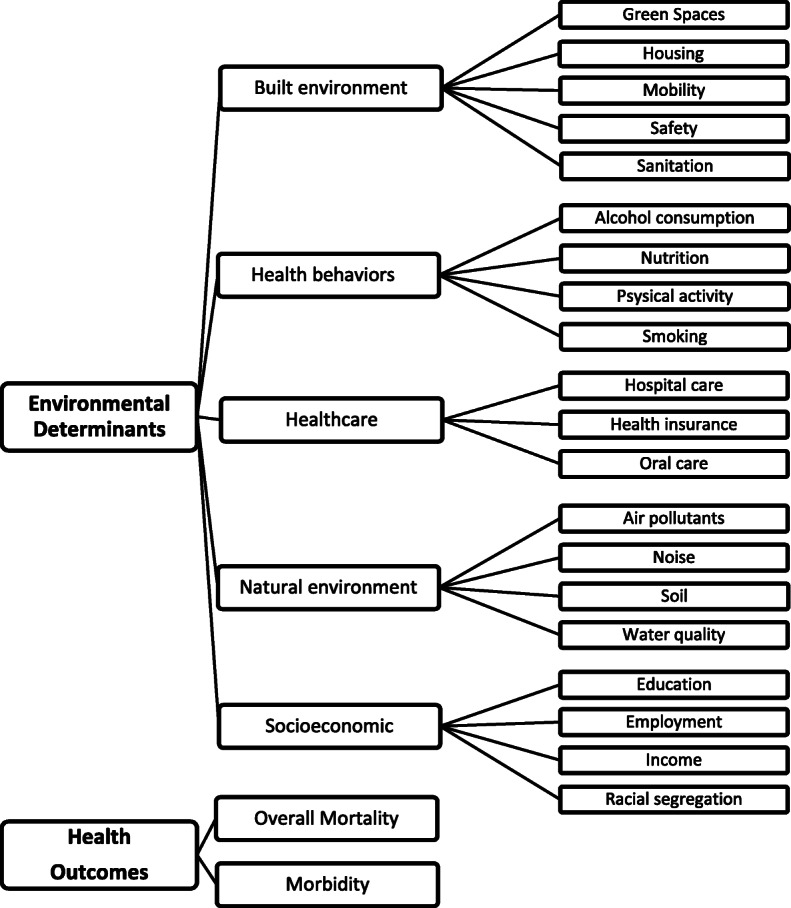
Illustrative representation of environmental determinants to evaluate population health in urban settings, adapted from [28–30]. The common dimensions were organize within the considered determinants with differences in: i) education and racial segregation are included in socioeconomic determinants; ii) physical environment is named natural environment, and the dimensions divided in air quality, water quality, noise and soil instead of natural resources; iii) built environment included green spaces and iv) behaviors included physical activity

This review was conducted according to the recommendations from the Preferred Reporting Items for Systematic Reviews and Meta-Analysis (PRISMA) [27]. A systematic search of PubMed, Web of Science, Scopus and SciELO Portugal database was conducted. Bibliographies of included articles were also searched for possible relevant articles (using the article title). Articles were eligible if they reported a relationship between at least one indicator operationalizing a dimension relevant for an environmental determinant (with socioeconomic, built environment, natural environment, health behaviors and/or healthcare determinants being considered) and at least one indicator operationalizing an health outcome (entailing all causes mortality and/or morbidity) in urban settings, areas with high density of population and build-up area [11]. In the adopted nomenclature, determinant – e.g. natural environment - is divided into dimensions like air quality and noise, which are then operationalized through indicators, such as, concentration of particulate matter (PM) or day-evening-night level (L_den_). A representation of the environmental determinants and dimensions relevant to evaluate population health in urban settings is depicted in Fig. 1.

**Fig. 2:**
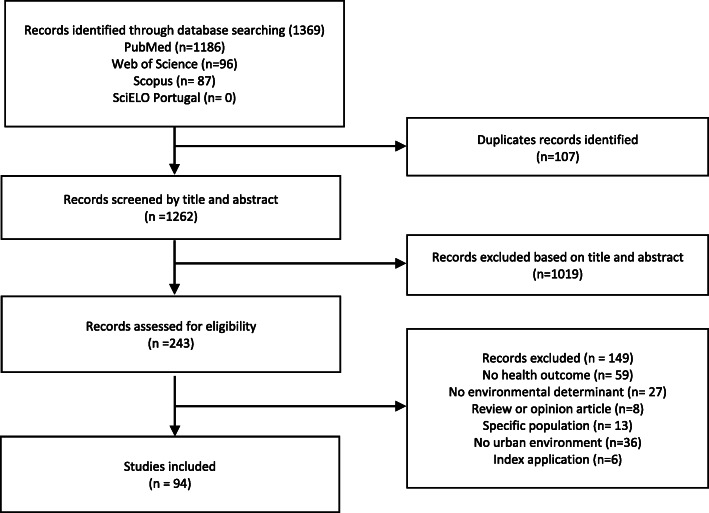
PRISMA flow chart with literature search

### Search strategy

The period covered in the search was from 2008 to 2018 and the following syntax was used: (#1) (“population health”[All fields]) AND (#2) (city OR cities OR town OR “metropolitan area” OR “urban environment”[Title/Abstract]) AND (#3) (indicators OR determinants [Title/Abstract]).

As inclusion criteria each study had to: (1) be written in English or Portuguese; (2) report a quantitative relationship between at least one environmental determinant, and one health outcome and (3) population health should be analyzed in urban settings at city level, council or metropolitan area (studies performed in Brazil municipalities must state if the municipality is an urban environment).

The exclusion criteria were: (1) specific populations as migrants or indigenous populations or population living in slums; (2) genetic studies or studies using animal models, as well as studies evaluating the applications of tools or indexes and studies comparing rural and urban environments; (3) qualitative studies, systematic reviews and meta-analyses and (4) studies that were only published in abstract form. Although, we recognize the value of grey literature, in the current systematic review this form of publication was not considered due to potential risk of bias.

### Study selection and data extraction

Two authors (MS and JM) independently screened all included titles and abstracts of the entire list of studies identified and reviewed full texts of articles that met predetermined inclusion criteria.

All the references identified through the search were uploaded into citation manager software ENDNOTE (X7, Thomson Reuters) and duplicates were removed. Data extracted for each publication was organized by environmental determinant, grouped by category of heath outcome and included: author and date, aim of the study, study population, study design, association measure, dimension and respective indicators, and type of relation between indicator and health outcome (Additional file 1).

The visualization of the relationships between environmental determinants dimensions and health outcomes evidenced in the extracted data was made using Sankey diagram (http://sankeymatic.com/build/). Key conclusions and policy recommendations were extracted to inform the construction of a final framework to analyze environment related population health in urban settings. Discrepancies were solved through a review by a third coauthor (AM).

### Quality assessment

Acknowledging the relevance of assessing the quality of studies, we evaluated the risk of bias of the sampled studies by means of a checklist previously used in reviews assessing the impact of environmental determinants on health [31, 32]. For each study, two investigators (MS and JM) independently evaluated the risk of bias associated with exposure assessment, confounding, selection of participants, and health outcome assessment, leading to a risk classification for each bias and globally (low, high and unclear). The studies that did not obtain the same risk of bias class from the two investigators were discussed with the third author (AM) to reach consensus. The classes set in [31] and the respective assessment of the sampled studies are shown in Additional file 2.

## Results

The literature search identified 1369 records. After removing the duplicate records, 1262 studies were screened based on title and abstract and 1019 records were excluded, leaving 243 articles for full-text screening. Ninety-four records met the inclusion criteria and were included in this review while 149 were excluded. Figure 2 provides the flow diagram of articles included and excluded from the review.

Environmental determinants were divided in socioeconomic status, natural environment, built environment, healthcare, and behaviors. Health outcomes were divided into 5 major categories: 1) overall mortality, 2) morbidity related to birth outcomes (low birth weight, preterm, low height and weight for gestational age), 3) morbidity related with overall chronic diseases outcomes (e.g. cancer, cardiovascular, impairment, HIV, oral diseases and respiratory) [33], 4) morbidity related with mental illness and 5) morbidity caused by obesity health conditions provides the flow diagram of articles included and excluded from the review.

Environmental determinants were divided in socioeconomic status, natural environment, built environment, healthcare, and behaviors. Health outcomes were divided into 5 major categories: 1) overall mortality, 2) morbidity related to birth outcomes (low birth weight, preterm, low height and weight for gestational age), 3) morbidity related with overall chronic diseases outcomes (e.g. cancer, cardiovascular, impairment, HIV, oral diseases and respiratory) [33], 4) morbidity related with mental illness and 5) morbidity caused by obesity health conditions.

Out of the sample of 94 studies, the largest number of included studies were published between 2012 and 2016. Predominantly the referred studies analyzed the impact of an environmental determinant and/or dimension making use of more than one indicator; and more than half focused on adult populations (18–64-years-old). Most of the studies had a cross-sectional (56%) and cohort (37%) design and the association measures were mainly odds ratio, relative risk, β coefficient and prevalence ratio.

**Fig. 3:**
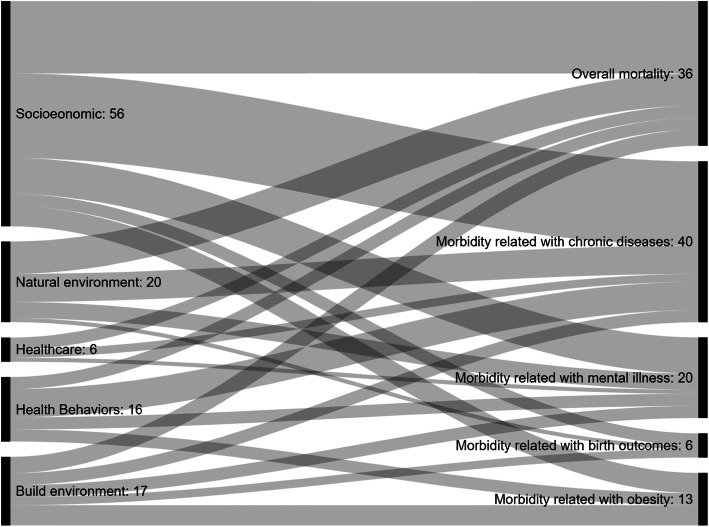
Sankey diagram of studies exploring relationships between environmental determinants and health outcomes (*N* = 94 studies)

The 94 studies explored 24-paired associations between 45 indicators (within 5 environmental determinants) and the 5 categories of health outcomes. The multilevel mapping Sankey diagram displayed in Fig. 3 shows the relationships between environmental determinants and the major categories of health outcomes of the 94 studies. The characteristics of each included study were systematically analyzed and summarized in Tables 1–10 (see Additional file 1), in which the relationship between the environmental indicator and health outcome was categorized as follows:

- positive (+), if a desirable improvement in the indicator was associated with an improvement of population health (i.e. a decrease in unemployment is associated with better health), or if a population subgroup is associated with higher population health (i.e. in case White has comparatively higher health than other groups);
- negative (−), if a desirable improvement in the indicator was associated with a deterioration of population health (i.e. a decrease in unemployment is associated with worse health), or if a population subgroup is associated with worse population health (i.e. in case a Black has comparatively worse health than other groups);

Such cases of positive or negative associations are presented in Tables 1, 3, 5, 7, 9 in Additional file 1. If the study reported an association not statistically significant (for the defined statistical level) between an indicator and the health outcome, it was categorized as null (0) (as in Tables 2, 4, 6, 8, 10 in Additional file 1). The published research was conducted in various locations with a high contribution of studies conducted in Europe (35%) followed by Brazil (26%) and USA (16%).

Looking into specific environmental health determinants, from the 57 studies evaluating the impact of socioeconomic determinant, 81 indicators showed association with population health. All improvements in socioeconomic determinant indicators were found to positively impact population health.

Of the 36 indicators used to understand the relationship between natural environment and population health, obtained from 18 studies, the evidence showed that increases in the quality of water and decreases in all air pollution and noise indicators are associated with improved on overall mortality, birth outcomes, chronic diseases (cardiovascular, cancer, and respiratory) and mental disorders.

Results from the 18 indicators of built environment show that improvements in mobility and green spaces would improve population health related with overall mortality, birth complications, chronic diseases, mental disorders and obesity outcomes. Sanitation and safety improvements are associated with improvements in birth outcomes, mental disorders, and obesity outcomes.

Only 5 studies assessed the healthcare determinant showing that increases in hospital supply and infrastructures have positive associations with overall mortality, while dental care use and health infrastructures showed null associations with any health outcome.

Among health behaviors determinant, from the 21 indicators referred in the 14 studies included, improvements in human behavior indicators translated into improved population health but no association was found with birth outcomes and morbidity related with HIV and respiratory diseases.

Contrasting with the initial framework defined for analysis, there was no study assessing the impact of soil indicators, housing indicators and health insurance indicators on mortality and morbidity outcomes. From the reviewed studies, 78% of the studies were found to have overall high risk of bias (Additional file 2), mostly because of a high bias due to blinded health outcome assessment.

**Fig. 4:**
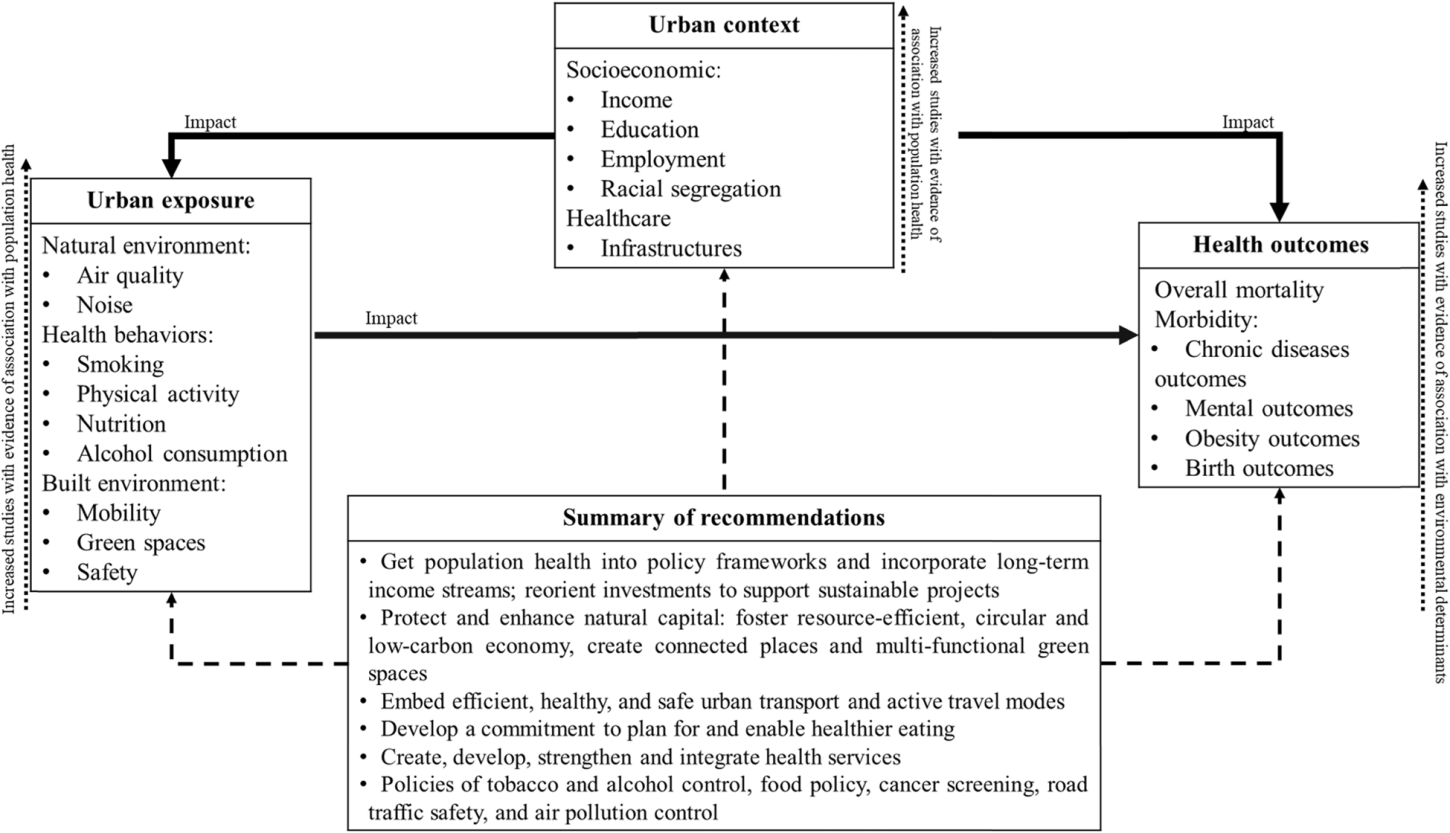
Summary of environmental determinants and dimension based upon the review, deemed as relevant for urban contexts, and synthesis of preventive recommendations to promote population health in urban contexts

Lastly, Fig. 4 systematizes the determinants and dimensions hierarchy relevant to analyze environmental population health in urban settings, based on findings of this review and on recommendations extracted from the studies included. The urban context and exposure boxes present environmental health dimensions ranked by evidence of association with health as captured by the number of studies providing evidence of association (dimensions without evidence of association were excluded). The health outcomes box displays the main outcomes dimensions relevant to measure environmental population health in urban settings. The straight arrows show generic impact associations.

## Discussion

### Sample of reviewed studies

This systematic review was performed to elucidate the nature and state of current evidence on the relationship between environmental determinants and indicators and health outcomes in urban settings. It was based on 94 studies with a clear heterogeneity of methodological approaches, targeted populations and association measures which can explain why a high percentage of studies entailed high risk of bias (78%) with risk of bias being mainly attributed to issues in outcome assessment. Most of the studies were performed with populations from USA, Brazil, and Europe. This predominance can be explained by the fact that the most urbanized regions include Northern America (82% living in urban areas in 2014), Latin America (80%), and Europe (73%). African and Asian countries remain mostly rural, with 40 and 48% of their respective populations living in urban areas [11].

Our strict inclusion criteria guaranteed that only studies assessing a clear relationship between an environmental determinant and an outcome were included. This was to objectively appraise that relationship, as well as the risk of bias and facilitate the interpretation of the evidence to increase validity of results and constancy across the data extraction.

### Evidence on environmental determinants and associations

The evidence presented in the studies included in this systematic review demonstrated the importance of understanding the complex interdependency of health, society, socioeconomic condition, built and natural environment [34–37], as well as an increasing consensus about the repercussions of surrounding environment on population health, and also on the specificities of environmental population health measurement in urban contexts.

The overall findings suggest that socioeconomic determinant have been the most studied area, evidencing strong and consistent associations with all health outcomes appraised in this review. Lorenzoni (2019) and Pickett (2015) [38, 39] shows that income inequality, measured mainly as median household income, has a strong impact on health what is aligned with the inverse associations found in this review, that indicate that improvements in indicators like income, education, employment status and racial inclusion, could result in a reduction in mortality and morbidity outcomes improving overall population health. Indeed, lower mortality and morbidity rates among socioeconomically advantaged people have been observed for hundreds of years, and in recent decades these observations have been replicated using various indicators of socioeconomic (percentage of people working or ranking like blue vs white collar) status and while considering multiple disease outcomes. A careful analysis of the results revealed an additive influence on the impact of these indicators with the outcome, meaning that improving more than one indicator simultaneously could result in a higher improvement on health. From a policy perspective, as well as from an etiological perspective, it is important to understand which of the components is critical - for instance, if education is found to be important, the policies that may be implemented would differ from the policies needed if income was found to be the most influential factor. In fact, most research has not tested such competing hypotheses directly, although the indicators used in each study are explicitly identified.

The constant need to monitor the state of the natural environment to check if the international targets are being achieved and if policy actions are having the desired effects [40] can explain natural environment determinant emerging as the second area with the most evidence on associations with population health. Evidence was found that improvements in ambient air pollution (PM2.5, PM10, NO_2_, SO_2_, O_3_, total suspended particles (TSP)) and noise levels (L_den_, L_night_) resulted in lower rates of mortality, as well as in decreased numbers of birth complications, chronic diseases such as cardiovascular, cancer and respiratory, and mental outcomes. In general, the studies reviewed evaluated separately the impact of air pollution and noise on health supporting the evidence that environmental noise should be considered an independent risk factor to health separated from air pollution [41, 42]. Another perspective shows that there is a relationship between air pollution and noise generated by traffic road traffic in cities [43]. In fact, depending on which health outcome is being analyzed and which types of pollutants are being measured the effect could be independent or cumulative. These perspectives should lead to the adoption of common measures for each category of health outcomes and of common mitigation strategies in urban environments. It was not found any evidence relating soil quality indicators and health, in urban settings. The restriction to cities, where agriculture has few expression in daily life can explain the lack of evidence or as mention by Morrison (2014) [44] there is a link between soil and air pollutants, but the associations between air quality and health are more pronounced. The studies assessing the impact of built environment indicators on health are heterogeneous. This could be related to variations in measures and tools used across studies, making difficult to compare findings and obtain uniform results [45]. There has been a weak evidence that improving built environment indicators is associated with improvement of health outcomes, but it is necessary more information to infer a causal relation between them [46]. Within a context of increasing urbanization, urban green spaces are gaining a growing interest for their role as an important element for sustainable and healthy societies in an urban context [47]. Green spaces contributes to the urban ecosystem through air purification, water and climate regulation, reduce air pollution by absorbing certain airborne pollutants from the atmosphere, biodiversity, providing benefits to urban residents (recreation, social interaction and inclusion, health benefits and wellbeing), produces economic value by improving the quality of landscapes and the attractiveness of the city within the context of increasing competition [48, 49]. Additionally, green areas, including urban gardening, parks and other natural areas, have been associated with lower stress scores, decrease of obesity rates [50], increased physical activity, and improved well-being and health in general [31, 51, 52]. No study proved that housing conditions have a relation with health outcomes, and sanitation indicators was analyzed only by Cau (2016) and de Souza (2012) [53, 54] showing that increasing wastewater treatment and quality of drinking water are associated with mental health improvements.

The evidence about relationship between behavior indicators and population health shows many positive associations, especially in studies in which improvements in more than one indicator of behavior were analyzed - improvements in behavior-related indicators should improve health outcomes like mortality, chronic diseases, mental and obesity disorders.

Lastly, a scarce number of studies reported the relationship between healthcare indicators and mortality outcomes - evidence was reported only in American and Brazilian populations and showed a positive association between improvements in hospital care and improvements on population health.

Jia (2017) [55] suggests that the role of health behaviors and healthcare indicators are tied to demographic characteristics and socioeconomic inequality, acting as an indirect pathway with impact on health and the results of this review can be explained by this. As a healthcare determinant works as a mediating pathway of inequality to mortality, the evidence about the association of healthcare indicators and population health is limited.

### Evidence on health outcomes

Overall mortality and chronic diseases morbidity were the most studied outcomes in the reviewed studies. Associations with all the determinants evaluated were found, as well as for mental outcomes. Obesity outcomes appear as the fourth health consequence in population health more influenced by environmental determinants, followed by birth outcomes. Within all the outcomes included in chronic diseases, HIV indicators were referred to be influenced only by socioeconomic indicators. Given that, the number of individuals newly infected with HIV has declined over the years but some groups remain at high risk [56], this can be an explanation for the present evaluation of the impact of environmental determinants on HIV indicators. However, the small number of studies may act as a bias and an indication that using HIV indicators to evaluate population health should be carefully discussed considering the specific urban context under analysis.

Evidence also showed that different measures to assess overall morbidity were used among the literature and in fact it may be a cofounding aspect that can generate divergences. To overcome this divergence, disability-adjusted life years (DALYs) or other health related quality of life metrics can be used to define and quantify the burden of disease, so as to measure the gap between current health status and an ideal situation free of disease and while combining mortality and morbidity indicators [57].

### Implications for environmental health analyses and policies in urban settings

The health of people living in cities is deeply determined by their living conditions. While there are considerable inequalities across regions, there are also inequalities within cities among various dimensions. The health challenges that need to be tackled to reduce population health inequities in urban environments are different from the ones found in rural environments in terms of, for example, air quality or access to health infrastructures, and must be analyzed differently [58]. These geographic differences reinforce the need for a differentiated environmental health assessment, using the right indicators and determinants to evaluate population health in urban environments and improve equity [59, 60]. Acknowledging the complexity and interconnectedness of population health assessment and their specificities for urban contexts, the purpose of collecting data related to determinants of population health in cities was to facilitate more evidence-based, rational, and prioritizing policy making.

Our results from the framework (Fig. 4) are consistent with the fact that health policies of tobacco control, alcohol control, food policy, and air pollution control have made significant contribution to advances in population health over the past decades, and remain an integral part of the political decision-making process in the context of urban settings [21]. To improve the link between evidence and policy actions, an extra box is added with recommendations measures [61–67] which are also aligned with recommendations from recent international reports and studies [68–72] for urban settings to promote population health.

### Strengths and limitations

This review has several strengths. As the main aim of this review is to report on associations, not to prove or refute causality, it presents an analysis of a wide and exhaustive range of influences between environmental determinants and health outcomes in urban settings. This urban settings focus enables an up to date identification of potential risks to population health. Although 25% of the reviewed studies were from Portuguese-speaking countries, the limitation to only select English and Portuguese written studies could have limited the evidence appraised in this review and to introduce a geographic location bias. Also, cities are spatially dynamic and can include suburban areas or slums contributing to the inherent complexity in mapping and in evaluating the quality of the heterogeneous data. Different populations, different methods and measures of evaluation, from the included studies and the variability of existing definitions of each determinant/dimension and outcome that were not standardized as might be expected may contribute to a misclassification bias. This heterogeneity should be considered when interpreting the high risk of bias of the reviewed studies. To minimize these issues, only peer review publications were included, and grey literature was excluded for the analysis.

## Conclusions

Our results provide a comprehensive synthesis of environment health determinants and indicators, outcomes and of associations between determinants and outcomes in urban settings, as well as identifies important gaps and methodological limitations in this field of research. Environmental health indices should be redesigned to reach consensus on definitions and measurements and to be meaningful to planners, policymakers, and researchers.

Ultimately, this review helps to identify those aspects of a city that influences and contribute to improve population health and suggests a hierarchy of determinants where actions to improve them should be taken to promote population health in urban settings.

Future work should look to improve flexible tools capable of evaluate modifications in environmental health determinants related to population health taking into account the dynamic of the urban setting to help target action areas, allocate resources and provide information to improve interventions and policies and to support decision making about health services and urban planning policies.

## Supplementary information

**Additional file 1.**

**Additional file 2.**

## Data Availability

Data sharing is not applicable to this article as no datasets were generated or analyzed during the current study.

## Abbreviations

HIV: Human Immunodeficiency Virus
USA: United States of America
