# Supplementary material for: Environmental determinants of population health in urban settings. A systematic review
Source: BMC Public Health. 2020 Jun 3;20:853. doi: 10.1186/s12889-020-08905-0 (PMC7271472; doi:10.1186/s12889-020-08905-0)
Supplement: Supplementary file 1 — Additional file 1. [file 12889_2020_8905_MOESM1_ESM.docx]

**Major determinants of population health in urban settings. A systematic review**

Marta Salgado^a^, Joana Madureira^b,c^, Ana Sofia Mendes^b,c^, Anália Torres^d^, João Paulo Teixeira^b,c^ and Mónica Oliveira^e^

^a^Institute of Environmental Health of Faculdade de Medicina, Universidade de Lisboa, Lisboa, Portugal; ^b^National Institute of Health, Environmental Health Department, Porto, Portugal; ^c^EPIUnit - Instituto de Saúde Pública, Universidade do Porto, Porto, Portugal; ^d^Valorsul S.A., São João da Talha, Portugal; ^e^Centre for Management Studies of Instituto Superior Técnico (CEG-IST), Universidade de Lisboa, Lisboa, Portugal.

| **Table of contents** | |
| --- | --- |
| ***Title*** | ***Page*** |
| Table 1 Studies of socioeconomic dimensions and analysed indicators and health outcomes with evidence of association | 2 |
| Table 2 Studies of socioeconomic dimensions and analysed indicators and health outcomes without an association not statistically significant (for the defined statistical level) | 25 |
| Table 2 Studies of built environment dimensions and analysed indicators and health outcomes with evidence of association | 31 |
| Table 2 Studies of built environment dimensions and analysed indicators and health outcomes without an association not statistically significant (for the defined statistical level) | 36 |
| Table 5 Studies of natural environment dimensions and analysed indicators and health outcomes with evidence of association | 39 |
| Table 6 Studies of natural environment dimensions and analysed indicators and health outcomes without an association not statistically significant (for the defined statistical level) | 46 |
| Table 7 Studies of healthcare dimensions and analysed indicators and health outcomes with evidence of association | 48 |
| Table 8 Studies of healthcare dimensions and analysed indicators and health outcomes without an association not statistically significant (for the defined statistical level) | 49 |
| Table 9 Studies of health behaviors dimensions and analysed indicators and health outcomes with evidence of association | 50 |
| Table 10 Studies of health behaviors dimensions and analysed indicators and health outcomes without an association not statistically significant (for the defined statistical level) | 56 |

Table 1 Studies of socioeconomic dimensions and analysed indicators and health outcomes with evidence of association

| **Health outcome** | **Reference** | **Overall aim** | **City/Cities Country** | **Study population** | **Study design** | **Health outcome measure** | **Association measure** | **Dimension/ Indicator** | **Relation between indicator and PH** |
| --- | --- | --- | --- | --- | --- | --- | --- | --- | --- |
| **Overall Mortality** | Belon, A. P. (2012) | To identify the magnitude of per  capita income differences in mortality rates among adults’ residents in a city of one million people. | Campinas; Brazil | Adults  (+20 years old) † | Ecological | Age adjusted mortality rates  *(years)* | Rate ratios | Income  *Monthly income*  *Men*: RR=1.17; 95%CI= 1.13, 1.20  *Women:* RR=1.30; 95%CI=1.25, 1.34 | + |
|  | Borrell, C. (2014) | To explore inequalities in total mortality and socioeconomic indicators between men and women in small areas of 16 European cities | Finland, Sweden, Belgium, France, Netherlands, UK, Switzerland, Czech Republic, Slovakia, Hungary, Italy, Portugal, Spain | Adults  (+25 years old)  *n=* *26,229,104 inhabitants* | Cross-  sectional ecological | Standardized mortality ratios (SMR) | Relative risk | Education and occupation  *Deprivation index (%) for men*  *Finland:* RR=1.144; 95%CI=1.108, 1.181  *Sweden:* RR=1.194; 95%CI=1.170, 1.219  *Belgium:* RR=1.022; 95%CI=1.011, 1.032  *France:* RR=1.060; 95%CI=1.035, 1.086  *Netherlands:* RR=1.037; 95%CI=1.020, 1.054  *UK:* RR=1.050; 95%CI= 1.043, 1.058  *Switzerland:* RR=1.051; 95%CI= 1.022, 1.081  *Czech Republic:* RR=1.227; 95%CI=1.020, 1.455  *Slovakia:* RR=1.101; 95%CI=0.975, 1.239  *Hungary:* RR=1.137; 95%CI=1.103, 1.174  *Italy:* RR=1.055; 95%CI=1.048, 1.062  *Portugal:* RR=1.058; 95%CI=0.991, 1.130  *Spain*: RR=1.046; 95%CI=1.039, 1.052 | + |
|  | Ceccon, R. F.  (2014) | To correlate suicide mortality and work indicators in six Brazilian metropolises. | Porto Alegre, Recife, Salvador, Belo Horizonte, Rio de Janeiro, São Paulo;  Brazil | Adults (+18years old)† | Ecological | Number of suicides | β coefficient | Employment  *% of employed population*  β=0.014; 95%CI= 0.006, 0.022 | + |
|  |  |  |  |  |  |  |  | Income  *% of people with income less then minimum wage per month*  β=-0.005; 95%CI=-0.383, 0.373 | + |
|  | Cheng, E. R. (2012) | To describe the association premature mortality among counties with broadly differing levels of income. | 3,139 USA counties | All individuals (to 75 years old) † | Cross-sectional* | All-cause, age-adjusted mortality rate per 100,000 population | β coefficient | Income  *Median household income ($)*  β=0.01, SE=0.02 | + |
|  |  |  |  |  |  |  |  | Racial segregation  *% nonwhite/nonblack race*  β=0.003, SE=0.005 |  |
|  | de Sousa Gdos, S. (2014) | To analyze the possible relationship income and education indicators with homicide mortality. | Fortaleza; Brazil | All individuals  *N=* *35,266 deaths of which 1,815 were victims of homicide* | Cross-sectional ecological | Rate of mortality by homicides per 100,000 inhabitants | β coefficient | Education  *Average years of study*  β=-0.001; p=0.141 | + |
|  |  |  |  |  |  |  |  | Income  *Per capta income*  β=-9.02; p=0.049 |  |
|  | James, W. L. (2012) | To understand if overall and race-specific mortality rates are combined with local health infrastructure data, income inequality and racial segregation. | 48 USA states | All individuals† | Ecological | Race-specific age/sex adjusted mortality rates per 100,000 | Moran’s I coefficient | Income  *Household income*  *Whites*: I=0.007; p<0.001  *Blacks*: I=0.009; p<0.05 | + |
|  |  |  |  |  |  |  |  | Racial segregation  *Percentage of Black*  *Whites*: I=0.238; p>0.05  *Blacks*: I=0.430; p>0.05 |  |
|  | Lee, J.  (2014) | To examine if adult mortality from injuries in South Korean metropolitan cities is affected adjusting for socioeconomic indicators. | Seoul, Busan, Incheon, Daegu, Gwangju, Daejeon, Ulsan;  South Korea | Adults  (≤34 years old)  *n=10 583859* | Cross-sectional* | Number of deaths by suicide, traffic accidents, falls and all injuries | Risk ratio | Education  *Worst deprivation index*  *Traffic accidents*: RR=1.34; 95%CI= 1.05, 1.73  *Falls*: RR=1.63; 95%CI=1.20; 2.20  *Suicide*: RR=1.09; 95%CI= 1.01; 1.17  *All injuries*: RR=1.14; 95%CI= 1.07; 1.22 | + |
|  | Nolasco, A. (2015) | To describe inequalities in preventable avoidable mortality in relation to socioeconomic status in small urban areas of thirty-three Spanish cities. | Spain | All individuals† | Ecological | Number of deaths | Relative Risk | Education and Occupation  *Socioeconomic status*  *0-44years old:* RR=3.0; SE=0.7 (*SES1 vs SES3*)  *45-64years old:* RR=2.3; SE=0.8 (*SES1 vs SES3*)  *>64years old:* RR=1.7; SE=0.5 (*SES1 vs SES3*) | Men: + |
|  | Nolasco, A. (2014) | To describe inequalities in preventable and amenable mortality in relation to socioeconomic status in 3 small urban areas. | Alicante, Castellón, Valencia; Spain | All individuals (0-74 years old)† | Transversal ecological | Frequency of amenable deaths in non-institutionalized individuals | Relative Risk | Education and Occupation  *Socioeconomic status*  Alicante *(SES3)*  *Men*: RR=1.5; 95%CI=1.1, 1.9  *Women*: RR=1.8; 95%CI=1.3, 2.4  Castellón *(SES3)*  *Men*: RR= 1.4; 95%CI= 0.9, 2.1  *Women*: RR=1.7; 95%CI=1.7; 1.1, 2.6  Valencia *(SES3)*  *Men*: RR=1.4; 95%CI=1.2, 1.6 | + |
|  | Ribeiro, A. I. (2016) | To evaluate the spatial distribution of old-age survival and its relationship with built environment and deprivation. | Porto;  Portugal | Elderly  (75–84 years old)† | Cohort* | Old-age survival | Odds ratio | Income  *European Deprivation Index*  *Men*: OR=1.31; 95%CI= 1.05, 1.63  *Women:* OR=1.53; 95%CI= 1.24, 1.89 | + |
|  | Sanchez-Barriga, J. J. (2012) | To determine and to establish an association between education with mortality from hypertension. | 31 Mexico states | Adults  (+18 years old)† | Ecological | Age-adjusted mortality rates nationwide per 100 000 inhabitants | Relative Risk | Education  *Education level*  *Incomplete elementary school:* RR=1.462; 95%CI= 1.442, 1.482  *Complete elementary school:* RR= 0.251; 95%CI= 0.245, 0.257  *High school or equivalent:* RR= 0.171; 95%CI= 0.167, 0.176  *Senior in high school or equivalent:* RR= 0.098; 95%CI= 0.095, 0.102  *College:* RR= 0.121; 95%CI= 0.117, 0.125 | + |
|  | Santos, S. L.  (2016) | To identify factors associated with infant mortality by a hierarchical model based on socioeconomic determinants like maternal education and maternal occupation. | Teresina; Brazil | Newborns (Mother age: +10 years old)  *n=13,882* | Cohort | Number of love births and numbers of death births | Odds ratio | Education  *Education level*  *Low:* OR=1.85; 95%CI=1.43, 2.32  *Intermediate:*1, p< 0.001  *High:* OR=1.28; 95%CI=1.10, 1.47 | - |
|  |  |  |  |  |  |  |  | Employment  *Mothers occupation*  *With remuneration: OR= 2*.03; 95%CI=1.48, 2.44 |  |
|  | Vandenheede, H.  (2014) | To quantify and compare socioeconomic inequalities measured by education level and deprivation, in all-cause mortality in urban population samples from 10 cities. | Czech Republic, Russia, Poland, Lithuania | Adults  (45–69 years)  *n=2750* | Cohort | Number of all-causes deaths | Hazard ratios | Education  *Education level*  *Lower secondary:* HR=1.8; 95%CI=1.5, 2.1  *Upper secondary:* HR=1.4; 95%CI=1.3, 1.6 | Men: + |
|  |  |  |  |  |  |  |  | Income  *Income deprivation*  *Yes:* HR=1.4; 95%CI=1.2, 1.6 |  |
|  | Walsh, D. (2010) | To analyze the relation between income deprivation and overall mortality. | Glasgow, Liverpool, Manchester; UK | All individuals† | Cohort* | Number of deaths | Standardized mortality ratio | Income  *Scottish Index of Multiple Deprivation (SIMD)*  SMR=114.4; 95%CI=113.2, 115.5 | + |
| **Morbidity related with birth outcomes** | de Souza, O. F.  (2012) | To investigate the prevalence of malnutrition associated with socioeconomic conditions, access to services and childcare. | Acre;  Brazil | Children  (<5 years old)  *n=* *667* | Cross-sectional | Prevalence  by height for age  and weight for height deficits | Prevalence ratio | Education  *Education level*  *Having an illiterate father or stepfather:* PR=1.82; 95%CI=1.01, 3.27 | + |
|  |  |  |  |  |  |  |  | Income  *Household wealth index*  PR=1.74; 95%CI=0.95, 3.18 |  |
|  | Garcia-Subirats, I. (2011) | To describe economic inequalities in low birth weight, preterm birth and small for gestational age births, in urban neighborhoods. | Barcelona; Spain | Newborns (Mother age: 12–49 years old)  *n=192,921 live births* | Cross-sectional ecological | Prevalence of  Low birth weight, Preterm  Small for gestational age births | β coeficient | Employment  *Unemployment rate*  *Low birth weight*: β=0.10; 95%CI= 0.07, 0.12  *Preterm*: β=0.06; 95%CI= 0.04, 0.08  *Small for gestational age*: β=0.10; 95%CI= 0.07, 0.12 | + |
|  | Hayward, I.  (2012) | To investigate if socioeconomic status might be viable target of interventions to reduce differential risk of small gestational age. | Vancouver; Canada | Newborns (Mother age:20-35 years old)  *n=59,039 live, singleton births* | Cross-sectional* | Small gestation age (SGA) | Odds Ratio | Income  *Average income quintile*  *($24,444–$28,440)*: OR=0.9; 95%CI=0.8, 1.0  *($28,440–$32,954)*: OR=0.9; 95%CI=0.8, 1.0  *($32,986–$38,832):* OR=0.8; 95%CI=0.7, 0.9  *($38,837–$509,269)*: OR=0.8; 95%CI=0.7, 0.8 | + |
| **Morbidity related with chronic diseases** | Banerjee, D. (2010) | To determine the role of education in the  association of functional status, chronic disease and  civic participation with SRH. | Houston; USA | Elderly  (>60 years old)  *Weighted*  *n= 127* | Cross-sectional | Self-reported health (SRH)  *% prevalence of chronic conditions* | Odds Ratio | Education  *Literacy level*  *Arthirtis*: OR=1.99; 95%CI=0.92, 4.30  *Diabetes*: OR=2.12; 95%CI=0.93, 4.81  *Hypertension*: OR=4.29; 95%CI=1.85, 9.98 | + |
|  | Buot, M. L. (2014) | To examine the relationship between income inequality, poverty, educational attainment, residential segregation and HIV incidence across eighty large cities. | USA | Adults  (+18 years old)† | Cohort* | Average HIV incidence | HIV Association Factor | Education  *High School level or More*  HIV=1.51; 90%CI= 1.17, 2.00 | + |
|  |  |  |  |  |  |  |  | Employment  *% unemployment*  HIV=1.76; 90%CI= 1.42, 2.17 |  |
|  |  |  |  |  |  |  |  | Income  *Median household income*  HIV=1.49; 90%CI= 1.20, 1.86 |  |
|  |  |  |  |  |  |  |  | Racial segregation  *Black isolation*  HIV=1.73; 90%CI= 1.40, 2.16 | - |
|  | Cabral, D. M. (2014) | To determine the prevalence of chronic pain and to identify sociodemographic, psychosocial, and occupational factors associated. | São Paulo;  Brazil | Adults  (+15 years old)  *n=* *1,108* | Cross-sectional | Chronic Pain Grade (CPG) | Prevalence ratio | Employment  *% unemployment*  PR=1.78; 95%CI=0.81, 3.91 | + |
|  |  |  |  |  |  |  |  | Education  *≤4 Years* *of study*  PR=1.28; 95%CI=1.09, 1.51 |  |
|  | Cordoba-Dona, J. A.  (2012) | To determine the relationship between  HIV-TB and non-HIV-TB incidence and social deprivation indicators. | Campo de Gibraltar; Spain | All individuals† | Cross-sectional | Tuberculosis incidence by HIV  status | Incidence rate ratio | Education and Occupation  *Socioeconomic deprivation (Level 2)*  *HIV-TB*: IRR=1.32; 95%CI=0.73, 2.37  *Non-HIV-TB*: IRR=1.25; 95%CI=0.79, 1.97 | + |
|  | Enroth, L. (2013). | To investigate socioeconomic differences in health and functioning among nonagenarian men and women. | Tampere; Finland | Elderly  (+90 years old)  *n=1283* | Cross-sectional | Functional ability, comorbidity, and SRH indicators | Prevalence ratio | Education  *% high educated*  *Functional ability*: PR=3.46; 95%CI=1.59, 7.53  *Comorbidity*: PR=4.28; 95%CI=1.93, 9.47  *SRH*: PR=1.66; 95%CI=0.56, 4.91 | + |
|  |  |  |  |  |  |  |  | Employment  *% Upper nonmanuals*  *Functional ability*: PR=3.19; 95%CI=1.28, 7.98  *Comorbidity*: PR=2.39; 95%CI=0.96, 5.83  *SRH*: PR=1.86; 95%CI=0.50, 6.93 |  |
|  | Ferreira-Junior, O. M.  (2015) | To assess prevalence of dental pain and associated contextual factors. | 27 capital cities  Brazil | Children  (5 years old)  *n=7280* | Cross-sectional | Dental pain in the last 6 months | Prevalence rate | Education  *% incomplete primary education:* PR=1.03; 95%CI= 1.01, 1.06 | + |
|  |  |  |  |  |  |  |  | Income  *Per capita family income (U$)*  *≤218:* PR=2.67; 95%CI=1.33, 5.32  *219–656:* PR=2.11; 95%CI=1.03, 4.32  *657–1093:* PR=1.22; 95%CI=0.49, 3.07 |  |
|  |  |  |  |  |  |  |  | Racial segregation  *Black:* PR=1.10; 95%CI=0.88, 1.38  *Brown:* PR=1.20; 95%CI=*0.91, 1.56*  *Yellow (Asian descendants):* PR=0.63; 95%CI=*0.26, 1.57*  *Indigenous:* PR=1.97; 95%CI=1.19, 3.26 |  |
|  | Fleischer, N. L.  (2008) | To investigate the associations of individual- and area-level SES with chronic disease risk factors. | Buenos Aires; Argentina | Adults  (+18 years old)  *n=1510* | Cross-sectional | Percentage of  High blood pressure (diagnosed at least once) and  Diabetes (diagnosis) | Odds Ratio | Education  *Secondary or universitary level*  *Hypertension*: OR=1.48; 95%CI=0.99, 2.20 *Diabetes*: OR=4.12; 95%CI=1.85, 9.18 | + |
|  |  |  |  |  |  |  |  | Income  *Household monthly income (in pesos)*  *Hypertension*: OR=1.50; 95%CI=0.99, 2.26 *Diabetes*: OR=2.43; 95%CI=1.14, 5.20 |  |
|  | Goulart, M. D.  (2016) | To assess if adults living in cities with a relative increase on income inequality were more likely to have severe tooth loss and lack a functional dentition. | Capital cities Brazil | Adults  (35-44 years old)  *n=6366* | Cross-sectional | Tooth loss outcomes | Odds ratio | Income  *Family monthly income (R$)*  *Severe tooth loss*  *1.501-2.500:* OR=1.09; 95%CI=0.65, 1.84  *501-1.500:* OR=1.42; 95%CI=0.89, 2.26  *<500:* OR=2.59; 95%CI=0.93, 2.70  *Lack of functional dentition*  *1.501-2.500:* OR=1.63; 95%CI=1.26, 2.12  *501-1.500:* OR=1.95; 95%CI=1.53, 2.48  *<500:* OR=2.54; 95%CI=1.92, 3.37 | + |
|  | Lacerda, J. T. (2008) | To analyze the relationship between oral health conditions in adult’s socio-demographic characteristics. | Santa Catarina; Brazil | Adults  (35-44 years old)  *n= 622* | Transversal | Oral Impacts on Daily Performances (OIDP) | Odds ratio | Education  *<8 Years of study*  OR=1.456; 95%CI= 0.76, 2.78 | + |
|  |  |  |  |  |  |  |  | Income  *Monthly household inccome until 3 minimum wages*  OR=1.49; 95%CI=0.75, 2.95 |  |
|  | Lopes, E. M. (2015) | To analyze the relationship between pediatric AIDS cases and neighborhood socioeconomic indicators. | Fortaleza; Brazil | Children (<12 years old)† | Ecological | Number of AIDS cases | Moran index | Income  *Monthly household income*  I=0,6; p= 0,001 | + |
|  | Morisco, F. (2017) | To assess the prevalence of risk factors for hepatitis C virus (HCV) infection in a large metropolitan area. | Naples;  Italy | Adults  (+18 years old)  *n=1315* | Cross-sectional | Prevalence of HCV | Odds Ratio | Education  *Low level: elementary/secondary education only*  OR=3.6; 95%CI=1.4, 9.3 | + |
|  | Pinto-Sarmento, T. C.  (2016) | To investigate socioeconomic factors associated with the presence of dental caries in preschool children. | Campina Grande; Brazil | Children  (3-5 years old)  *n=* *843* | Cross-sectional | Number of diagnosis of  dental caries and lesion activity | Odds ratio | Education  *≤8 Years of study*  OR=2.15; 95%CI= 1.15, 4.00 | + |
|  |  |  |  |  |  |  |  | Income  *Monthly household income*  *≤U$ 312.50:* OR=1.83; 95%CI=0.96, 3.50 |  |
|  | Piovesan, C. (2010) | To assess the inequality in caries distribution and the association between socioeconomic indicators and caries experience of preschool children. | Santa Maria; Brazil | Children  (1-5 years old)  *n=455* | Cross-sectional | Prevalence decayed, missing and filled primary teeth (dmf-t index) | Rate ratio | Education  *<8 Mother’s level of education (years)*  RR=2.03; 95%CI= 1.28,3.23 | + |
|  |  |  |  |  |  |  |  | Racial segregation  *% Non-white*  RR=1.71; 95%CI= 1.11, 2.81 |  |
|  | Pizzo, G. (2010) | To investigate the prevalence of caries in children and assessed the relationship between socio-behavioral determinants and caries. | Palermo; Italy | Children  (5 and 12 years old)  *n=511* | Cross-sectional* | Dmf-t index | Odds ratio | Education  *Mother’s educational level*  *Middle vs Primary*  OR=0.07, 95%CI=0.02, 0.35 | + |
|  | Ramsay, S. E. (2008) | To examine the determinants of socioeconomic inequalities in disability and functional limitation in elderly men. | 24 cities  United Kingdom | Elderly  (63-82 years old)  *n=3981* | Cross-sectional | Activities of daily living (ADLs) | Odds Ratio | Income  *Registrar Generals’ Social Class Classification*  *(class V vs I)*  *ADL*: OR=3.13; 95%CI=1.64, 5.97  *ADL disability*: OR=2.87; 95%CI=1.49, 5.51  *Functional limitation*: OR=2.65; 95%CI=1.31, 5.35 | + |
|  | Steer, S.  (2014) | To investigate the association between epilepsy and individual elements of deprivation in order to identify modifiable elements. | United Kingdom | Adults  (+18 years old)  *n=304,331* | Cohort* | Epilepsy prevalence (%) | Index of Multiple Deprivation | Education  *Education and training deprivation*  r=0.665, p<0.01 | + |
|  |  |  |  |  |  |  |  | Employment  *Employment deprivation*  r=0.629, p<0.01 |  |
|  |  |  |  |  |  |  |  | Income  *Income deprivation*  r=0.358, p<0.01 |  |
|  | Schulz, A. J. (2008) | To provide evidence of an effect linking socioeconomic indicators to cardiovascular disease (CVD) risk factors. | Detroit, Michigan; USA | Adults  (≥25 years old)  *n=919* | Cross-sectional* | Depressive symptoms, smoking status, physical activity,  body mass index and waist circumference | β coeficient | Income  *Monthly income ($)*  *Depressive symptoms:* β=-0,03, p<0.001  *Smoking status:* β=0.14, p<0.001  *Physical activity:* β=-0.01, p<0.001  *Body mass index:* β=0,26 p<0.001  *Waist circumference*: β =0.24, p<0.001 | + |
|  | Thorn, L. K. (2011) | To evaluate associations between areas with deprived socioeconomic conditions and pneumonia incidence. | Goiania; Brazil | Children  (<2 years old)  *n=11 521* | Prospective | Radiograph-confirmed pneumonia (CXR+Pn) | Incidence rate ratio | Education  *% of woman illiterate >10 years old*  IRR=1.119; 95%CI=1.032, 1.213 | + |
|  |  |  |  |  |  |  |  | Income  *% of head of households earning >20 minimum wages*  IRR=0.952; 95%CI=0.937, 0.967 |  |
|  | Trachtenberg, A. J.  (2014) | To examined whether differences in patient demographics, ambulatory care use, or physician characteristics could explain disparities in chronic obstructive pulmonary disease and asthma hospitalizations. | Manitoba;  Canada | Adults  (18-70 years old)  *n=34,741* | Cohort | Chronic obstructive pulmonary disease or asthma  grouped together as obstructive airway disease | Odds ratio | Income  *Income quintile*  *Q1:* OR=2.93; 95%CI=2.19, 3.93  *Q2:* OR=2.51; 95%CI=1.86, 3.40  *Q3:* OR=1.54; 95%CI=1.11, 2.13  *Q4:* OR=1.41; 95%CI=1.01, 1.98 | + |
| **Morbidity related with mental illness** | Aguilar-Palacio, I. (2012) | To identify the relationships between social factors and SRH. | Casablanca; Spain | Adults  (+15 years old)  *n=1032* | Cross-sectional | General  health index | Odds ratio | Education  *Primary or no education level*  *Vulnerable group:*  OR=1.00; 95%CI=0.99, 1.02 | + |
|  | Christiani, Y. (2015) | To examine unequally distribution of depression among women in Indonesia's major cities and investigate the factors contributed to the inequality. | Jakarta, Surabaya, Medan, Bandung; Indonesia | Adult  (19-65 years old)  *n=1117* | Cross-sectional* | Prevalence of depression | Concentration index | Income  *Monthly household expenditure*  CI=0.015, p<0.05 | + |
|  |  |  |  |  |  |  |  | Education  *Higher education level*  CI=0.27, p<0.05 |  |
|  | Faresjo, T. (2010) | To analyze the relations of social conditions to SRH status in the populations in white-collar city and a blue-collar twin city. | Norrköping, Linköping; Sweden | Adults  (20–64 years old)  *n=13,440* | Cohort* | SRH | Probability of no difference | Education  *High level (15 years or more)*  β=0.11; p=0.004 | + |
|  | Franca, M. H. (2016) | To estimate the association of prevalence rates of mental disorders with employment status. | São Paulo; Brazil | Adults  (+18 years old)  *n= 5037* | Cross-sectional | 12-month prevalence of 19 DSM-IV mental disorders | Odds ratio | Employment  *% Working:* OR=1.0  *% Inactive:* OR=2.8; 95%CI=1.51, 5.12  *% Unemployed:* OR=1.4; 95%CI=0.64, 3.14 | + |
|  | Habib, R. R. (2013) | To evaluate the association between women’s SRH and socioeconomic indicators. | Bebnine, Lebanon | Adult  (+14 years old)  *n=2223* | Cross-sectional | Self-reported health status | Odds Ratio | Education  *Read/write-secondary*  *Fair/Good:* OR=0.75; 95%CI=0.57, 0.99  *Poor/Good:* OR=0.51; 95%CI=0.30, 0.86  *Technical or university*  *Fair/Good:* OR=0.64; 95%CI=0.49, 0.83  *Poor/Good:* OR=0.24; 95%CI=0.12, 0.48 | + |
|  |  |  |  |  |  |  |  | Income  *Household income*  *Fair/Good:* OR=0.64; 95%CI=0.49, 0.83  *Poor/Good:* OR=0.24; 95%CI=0.12, 0.48 |  |
|  | Ribeiro Dos Santos, E. (2016) | To estimate the prevalence of depression associated with socio-demographic and economic characteristics, smoking habits, alcohol use, and physical morbidities. | Coari and Tefé;  Brazil | Adults  (+20 years old)  *n=34,838* | Cross-sectional | Score of ≥10 on the Patient Health Questionnaire-9 (PHQ-9) | Prevalence ratio | Income  *Personal monthly*  *income (R$)*  *500.00–1000.00*: PR=1.16; 95%CI=0.77, 1.74  *≤500.00*: PR=0.95; 95%CI=0.62, 1.46 | + |
|  |  |  |  |  |  |  |  | Education  *Educational level in years*  *9–11*: PR=1.13; 95%CI=0.77, 1.64  *5–8*: PR=1.11; 95%CI=0.75, 1.65  *0–4*: PR=1.35; 95%CI=  0.91, 2.00 |  |
|  | Scazufca, M. (2010) | To estimate the proportion of cases of dementia attributable to illiteracy, non-skilled occupation and low income. | São Paulo; Brazil | Elderly  (+65 years old)  *n= 2003* | Cohort* | Number of individuals diagnose with dementia | Population attributable fraction | Education  *% illiterate population*  PAF=9.4%; 95%CI=0.0%, 26.6% | + |
|  |  |  |  |  |  |  |  | Employment  *% people with non-skilled occupation*  PAF=29.2%; 95%CI=2.8%, 48.5% |  |
|  |  |  |  |  |  |  |  | Income  *% people with low income*  PAF=23.8%; 95%CI=6.9%, 37.7% |  |
|  | Sulander, T. (2012) | To examine the association of education and adequacy of income with SRH. | Helsinki; Finland | Elderly  (+75 years old)  *n=1395* | Cross-sectional | SRH | Cumulative odds ratios | Education  *Men (85+ years)*  *Middle school*: CORs=1.52; 95%CI=0.47, 4.98  *Elementary school*: CORs=1.23; 95%CI=0.40, 3.82  *Women (85+ years)*  *Middle school*: CORs=0.84; 95%CI=0.50, 1.42  *Elementary school*: CORs=1.70; 95%CI=1.01, 2.87 | + |
|  |  |  |  |  |  |  |  | Income  *Adquacy of income*  *Men (85+ years)*  *Average or less*: CORs=2.70; 95%CI=0.89, 8.17  *Women (85+ years)*  *Average or less*: CORs=3.24; 95%CI=1.99, 5.26 |  |
|  | Tucker-Seeley, R. D. (2013) | To determine the association between financial hardship and SRH. | Boston;  USA | Adults  (+18 years old)  *n= 828* | Cross-sectional | SRH | Relative risk | Education  *Educational level*  *Some high school:* RR=1.24; 95%CI=0.90, 1.72  *High school graduate:* RR=0.77; 95%CI=0.57, 1.03  *Greater than high school:* RR=0.65; 95%CI=0.50, 0.85 | + |
|  |  |  |  |  |  |  |  | Employment  *% Unemployed*  RR=1.29; 95%CI=1.03, 1.61 |  |
|  |  |  |  |  |  |  |  | Income  *% In poverty*  RR=1.16; 95%CI=0.94, 1.43 |  |
| **Morbidity related with obesity outcomes** | Akarolo-Anthony, S. N. (2014) | To examine the prevalence of overweight and obesity and its potential correlation with socio-economic status and occupation. | Abuja; Nigeria | Adults  (+18 years old)  *n=1058* | Cross-sectional | Body-mass index (BMI)  (kg/m^2^) | Prevalence ratio | Education  *Education level*  *≥Tertiary:* OR=1.13; 95%CI=0.92, 1.3  *None/Primary:* OR=1.39; 95%CI=0.30, 3.82 | + |
|  |  |  |  |  |  |  |  | Employment  *Professional/executive:* OR=1.00; 95%CI=0.86, 1.16  *Unskilled manual:* OR=0.84; 95%CI=0.59, 1.18  *Self-employed:* OR=0.79; 95%CI=0.38, 1.64 |  |
|  | Albaladejo, R.  (2014) | To assess whether the relationship between childhood obesity is explained by family socioeconomic position and risk behaviors. | Madrid; Spain | Children  (6–15 years old)  *n=* *727* | Cross-sectional* | Obesity (BMI)  (kg/m^2^) | Odds ratio | Education  *Percentage of population with tertiary studies*  *20.64-35.15:* OR=0.92; 95%CI=0.22, 3.86  *14.64-20.63:* OR=3.63; 95%CI=1.11, 11.87  *<14.64:* OR=3.42; 95%CI=1.00, 11.68 | + |
|  |  |  |  |  |  |  |  | Income  *Per capita income*  *€11,149.61-14,548.76*: OR=1.73; 95%CI=0.46, 6.49  *€9,724.29-11,149.60*: OR=3.10; 95%CI=0.91, 10.54  *< € 9,724.29:* OR=3.77; 95%CI=1.12, 12.70 |  |
|  | de Carvalho Cremm, E. (2012) | To investigate the individual and family determinants of being overweight among children living in an urban area. | Santos;  Brazil | Children (<10 years old)  *n=* *531* | Cross-sectional | BMI-for-age  (kg/m^2^) | Odds ratio | Education  *Mother’s education level*  *Completed high school:* OR=1.98; p=0.04 | + |
|  | Duarte-Salles, T. (2011) | To describe social inequalities in obesity among adolescents, by sex. | Barcelona; Spain | Children  (12-16 years old)  *n=* *903* | Cross-sectional | (BMI)  (kg/m^2^) | Prevalence ratio | Education  *Education level*  *Boys*  *Secondary:* PR=1.24; 95%CI=0.63, 2.44  *Primary:* PR=1.49; 95%CI=0.60, 3.68  *Girls*  *Secondary:*PR=1.66; 95%CI=0.78, 3.55  *Primary:* PR=3.30; 95%CI=1.34, 8.14 | + |
|  |  |  |  |  |  |  |  | Employment  *Family’s social class*  *Boys*  *Manuals:* PR=1.76; 95%CI=0.96, 3.23  *Girls*  *Manuals:* PR= 1.64; 95%CI= 0.87, 3.08 |  |
|  | Martin-Fernandez, J. (2014) | To determine whether food insecurity and obesity are associated. | Paris;  France | Adults  (+18 years old)  *n=2967* | Cross-sectional | (BMI)  (kg/m^2^) | Odds ratio | Education  *Education level*  *Men*  *Secondary*: OR= 2.77; 95%CI=1.70, 4.52  *None or primary*: OR= 2.38; 95%CI=1.17, 4.83  *Women*  *Secondar*y: OR= 1.24; 95%CI=0.85, 1.81  *None or primary*: OR=2.06; 95%CI= 1.21, 3.49 | + |
|  |  |  |  |  |  |  |  | Employment  *Women*  *Intermediate white-collar:* OR =1.18, 95%CI=0.65, 2.14  *Blue-collar*: OR =3.25, 95%CI=1.48, 7.14 |  |

Note: positive (+): a desirable improvement in the indicator was associated with an improvement of PH and negative (-): a desirable improvement in the indicator was associated with a deterioration of PH. *”Study design” assigned by the authors

Table 2 Studies of socioeconomic dimensions and analysed indicators and health outcomes without an association not statistically significant (for the defined statistical level)

| **Health outcome** | **Reference** | **Overall aim** | **City/Cities Country** | **Study population** | **Study design** | **Health outcome measure** | **Association measure** | **Dimension/ Indicator** | **Relation between indicator and PH** |
| --- | --- | --- | --- | --- | --- | --- | --- | --- | --- |
| **Overall Mortality** | Cheng, E. R. (2012) | To describe the association premature mortality among counties with broadly differing levels of income. | 3,139 USA counties | All individuals (to 75 years old) † | Cross-sectional* | All-cause, age-adjusted mortality rate per 100,000 population | β coefficient | Education  *% Adults with a 4-year college degree*  β=-0.04, SE=0.01 | 0 |
|  | Modrek, S. (2011) | To evaluate the relation between income inequality and mortality in the context of Costa Rica. | Costa Rica | Adults  (+15 years old)† | Longitudinal* | Deaths rate | Incidence rate ratios | Income  *Gini index*  IRR=0.978; 95%CI= 0.953, 1.004 | 0 |
|  | Nolasco, A. (2015) | To describe inequalities in preventable avoidable mortality in relation to socioeconomic status in small urban areas of thirty-three Spanish cities. | Spain | All individuals† | Ecological | Number of deaths | Relative Risk | Education and Occupation  *Socioeconomic status*  *0-44years old:* RR=2.4; SE=1.5 (*SES1 vs SES3*)  *45-64years old:* RR=1.3; SE=0.5 (*SES1 vs SES3*)  *>64years old:* RR=1.5; SE=0.9 (*SES1 vs SES3*) | *Women*: 0 |
|  | Rosicova, K. (2015) | To assess the associations between socioeconomic and ethnic neighborhood indicators and the all-cause mortality. | Bratislava, Kosice;  Slovak Republic | Adults  (20–64 years old)  *n=442,703* | Cross-sectional* | Standardized mortality per 100,000 inhabitants | Rate ratios | Education  *Education level*  *Low Education*: RR=1.004; 95%CI=0.998, 1.010 | 0 |
|  |  |  |  |  |  |  |  | Employment  *Unemployment rate:* RR=1.011; 95%CI=0.993, 1.029 |  |
|  |  |  |  |  |  |  |  | Income  *Income (in Euro’s):* RR=0.999; 95%CI=0.999, 1.000 |  |
|  | Unrath, M. (2014) | To analyze the influence of unemployment as indicator of neighborhood socioeconomic status on mortality in a stroke cohort. | Dortmund; Germany | Adults  (+18 years old)  *n=1883* | Cohort* | Age- and sex-adjusted mortality risks | Hazard ratios | Employment  *Quartiles of unemployment*  *First*: HR=0.51; 95%CI=0.27, 0.97  *Second*: HR=0.77; 95%CI=0.48, 1.25  *Third*: HR=0.82; 95%CI=0.54, 1.27 | 0 |
|  | Vandenheede, H.  (2014) | To quantify and compare socioeconomic inequalities measured by education level and deprivation, in all-cause mortality in urban population samples from 10 cities. | Czech Republic, Russia, Poland, Lithuania | Adults  (45–69 years)  *n=2750* | Cohort | Number of all-causes deaths | Hazard ratios | Education  *Education level*  *Lower secondary:* HR=1.8; 95%CI=1.4, 2.3  *Upper secondary:* HR=1.2; 95%CI=0.9, 1.4 | Women: 0 |
|  |  |  |  |  |  |  |  | Income  *Income deprivation*  *Yes:* HR=1.4; 95%CI=1.2, 1.7 |  |
| **Morbidity related with chronic diseases** | Ali, M. K. (2016). | To examined distribution of cardiovascular disease (CVD) risks across the socioeconomic spectrum, defined as education, wealth, and occupation. | Chennai, Delhi;  India  Karachi; Pakistan | Adults  (+20 years old)  *n=* *16,288* | Cross-sectional | Number of CVD risk factors | Age- and sex-standardized prevalence | Education  *Educational level (diabetes reference)*  *Up to Primary*: P=26%, p=0.008  *High/Secondary:* P=29%, p=0.008  *Graduate or higher:* P=25.5, p=0.008 | 0 |
|  |  |  |  |  |  |  |  | Income  *Household assets*  *Tertile Low:* P=26, *p<0.001*  *Tertile Medium:* P=28, *p<0.001*  *Tertile High:* P=30, *p<0.001* |  |
|  |  |  |  |  |  |  |  | Employment  *Not working*: P=28.5, *p<0.001*  *Semiskilled/Unskilled:* P=24.9, *p<0.001*  *Trained/ Skilled:* P=27, *p<0.001*  *White collar*: P=27.5, *p<0.001* |  |
|  | Bastos, L. N. V.  (2018) | To determine the incidence and association with environmental health indicators of cases of childhood and adolescent cancer. | Pernambuco; Brazil | Adolescents (<20 years old)  *n=* *1261* | Cross-sectional | Average Age-adjusted Incidence Rate of Cancer | Moran index | Income  *Gini index*  I=0.00161; P=0.017 | 0 |
|  | Cabral, D. M. (2014) | To determine the prevalence of chronic pain and to identify sociodemographic, psychosocial, and occupational factors associated. | São Paulo;  Brazil | Adults  (+15 years old)  *n=* *1,108* | Cross-sectional | Chronic Pain Grade (CPG) | Prevalence ratio | Income  *Family socioeconomic classification*  *B*: PR=1.11; 95%CI=0.67, 1.84  *C*: PR=1.12; 95%CI= 0.68, 1.86  *D*: PR=1.19; 95%CI=0.68, 2.07  *E*: PR=2.08; 95%CI=1.17, 3.69 | 0 |
|  | Schulz, A. J. (2008) | To provide evidence of an effect linking socioeconomic indicators to cardiovascular disease (CVD) risk factors. | Detroit, Michigan; USA | Adults  (≥25 years old)  *n=919* | Cross-sectional* | Depressive symptoms, smoking status, physical activity,  body mass index and waist circumference | β coeficient | Education  Education level (<High school)  *Depressive symptoms:* β=0.05, p<0.001  *Smoking status:* β=0.40, p<0.001  *Physical activity:* β=-0.32, p<0.05  *Body mass index:* β=-0.49, p<0.001  *Waist circumference*: β =-2.03, p<0.001 | 0 |
|  | Piovesan, C. (2010) | To assess the inequality in caries distribution and the association between socioeconomic indicators and caries experience of preschool children. | Santa Maria; Brazil | Children  (1-5 years old)  *n=455* | Cross-sectional | Prevalence decayed, missing and filled primary teeth (dmf-t index) | Rate ratio | Employment  *% Mothers unemployed*  RR=1.24; 95%CI=0.79, 1.95 | 0 |
|  |  |  |  |  |  |  |  | Income  *<3 Brazilian minimum wage*  RR=1.61; 95%CI=1.21, 2.75 |  |
|  | Pizzo, G. (2010) | To investigate the prevalence of caries in children and assessed the relationship between socio-behavioral determinants and caries. | Palermo; Italy | Children  (5 and 12 years old)  *n=511* | Cross-sectional* | Dmf-t index | Odds ratio | Employment  *Mother’s employment status*  *(data not showed)* | 0 |
| **Morbidity related with mental illness** | Christiani, Y. (2015) | To examine unequally distribution of depression among women in Indonesia's major cities and investigate the factors contributed to the inequality. | Jakarta, Surabaya, Medan, Bandung; Indonesia | Adult  (19-65 years old)  *n=1117* | Cross-sectional* | Prevalence of depression | Concentration index | Employment  *% people in paid work*  CI=0.005, p<0.0001 | 0 |
|  | Ribeiro Dos Santos, E. (2016) | To estimate the prevalence of depression associated with socio-demographic and economic characteristics, smoking habits, alcohol use, and physical morbidities. | Coari and Tefé;  Brazil | Adults  (+20 years old)  *n=34,838* | Cross-sectional | Score of ≥10 on the Patient Health Questionnaire-9 (PHQ-9) | Prevalence ratio | Racial segregation  *White (including Asian):*PR=1.20; 95%CI=0.82, 1.75  *Indigenous:* PR=1.06; 95%CI=0.62, 1.81 | 0 |
|  | Tucker-Seeley, R. D. (2013) | To determine the association between financial hardship and SRH. | Boston;  USA | Adults  (+18 years old)  *n= 828* | Cross-sectional | SRH | Relative risk | Racial segregation  *%* *Black non-Hispanic:* RR=0.74; 95%CI=0.48, 1.14  *% Hispanic:*  RR=0.85; 95%CI=0.55, 1.30  *%Other:*  RR=0.69 ;95%CI=0.42, 1.14 | 0 |
| **Morbidity related with obesity outcomes** | Martin-Fernandez, J. (2014) | To determine whether food insecurity and obesity are associated. | Paris;  France | Adults  (+18 years old)  *n=2967* | Cross-sectional | BMI  (kg/m^2^) | Odds ratio | Employment  *Men*  *Intermediate white-collar:* OR =0.67; 95%CI=0.33, 1.35  *Blue-collar*: OR =0.83; 95%CI=0.41, 1.68 | 0 |
|  |  |  |  |  |  |  |  | Income  *Income/consumption units €*  *3,000 – 4,500:* OR=1.64; 95%CI= 0.96, 2.83  *1,865–3,000*: OR=1.52; 95%CI=0.87, 2.67  *≤1,865:* OR=1.65; 95%CI=0.93, 2.94 |  |

*” Study design” assigned by the authors

Table 3 Studies of built environment dimensions and analysed indicators and health outcomes with evidence of association

| **Health outcome** | **Reference** | **Overall aim** | **City/Cities Country** | **Study population** | **Study design** | **Health outcome measure** | **Association measure** | **Dimension/ Indicator** | **Relation between indicator and PH** |
| --- | --- | --- | --- | --- | --- | --- | --- | --- | --- |
| **Overall Mortality** | Gronlund, C. J. (2015) | To examine how area characteristics independently modified the extreme heat-mortality association. | Michigan; USA | Elderly  (+65 years old) † | Time-stratified case-crossover | Number of primary causes of death | Odds ratio | Green spaces  *% non-green spaces*  *Low heat*: OR=0.97; 95%CI=0.89, 1.05  *Extreme heat*: OR=1.18; 95%CI=1.09, 1.28 | **+** |
|  | Habermann, M. (2012) | To assess the association between indicators of exposure to motor vehicle-related air pollution and cardiovascular mortality. | São Paulo; Brazil | Adults  (≥40 years old)  *n=9805* | Time-stratified case-crossover* | Mortality rates from cardiovascular diseases | β coefficient | Mobility  *Road density:* β= 0.096, p=0.017  *Light traffic volume:* β=0.0000237, p=0.703  *Heavy traffic volume:* β= 0.0000821, p=0.636  *Traffic volume:* β=0.0000212, p=0.663 | **+** |
|  | Harlan, S. L.  (2013) | To analyze neighborhood effects of population characteristics and built and natural environments on deaths due to heat exposure. | Arizona;  USA | Elderly  (+65 years old) † | Cross-sectional* | Number of heat-associated death | Odds ratio | Green Spaces  *Unvegetated area*  OR=1.19; 95%CI=1.02, 1.39 | **+** |
| **Morbidity related with birth outcomes** | de Souza, O. F.  (2012) | To investigate the prevalence of malnutrition associated with socioeconomic conditions, access to services and childcare. | Acre;  Brazil | Children  (<5 years old)  *n=* *667* | Cross-sectional | Prevalence  by height for age  and weight for height deficits | Prevalence ratio | Sanitation  *Exposure to open wastewater*:  PR=2.46; 95%CI=1.51, 4.00 | + |
|  | Grazuleviciene, R.  (2015) | To investigate whether surrounding greenness levels and/or distance to city parks affect birth outcomes in singleton live-births. | Kaunas; Lithuania | Newborns (Mothers age: +20 years old)  *n=3292 singleton live-births* | Cohort | Gestational age (GA, in weeks)  Preterm birth (PB, <37 gestational weeks)  Birth weight (BW, in g)  Low birth weight (LBW, birth weight below  2500 g)  Term low birth weight  (TLBW, birth weight below 2500g  Small for gestational age (SGA, birth weight below the 10th percentile) | Odds ratio | Green Spaces  *Normalized difference vegetation index* (*NDVI-500 ≤ median)*  *LBW*: OR=1.15; 95%CI=0.82, 1.61  *TLBW*: OR=1.92; 95%CI=1.29, 3.45)  *PB*: OR=0.81; 95%CI=0.59, 1.09  *SGA*: OR=1.03; 95%CI= 0.79, 1.33  *BW*: β=3.44; 95%CI=−23.4, 30.3  *GA*: β=0.09; 95%CI =−0.31, 0.22 | + |
| **Morbidity related with chronic diseases** | Dzhambov, A. M.  (2016) | To explore the overall association of prevalence of Type 2 diabetes mellitus (T2DM) with exposures to road traffic, noise and air pollution. | Plovdiv; Bulgaria | Adults  (+18 years old)  *n=* *513* | Cross-sectional | T2DM self-reported doctor diagnosis | Odds Ratio | Mobility  *Self-reported traffic intensity*:  *Moderate*: OR=1.15; 95%CI=0.30, 4.45  *High*: OR=1.40; 95%CI=0.48, 4.07 | + |
|  | Migliore, E. (2009) | To investigate if there were specific effects of cars and trucks traffic exposure on current asthma symptoms and cough or phlegm. | Turin, Milan, Rome;  Italy | Children and adolescents  (6–7/ 13-14 years old)  *n=33,632* | Cross-sectional* | Asthma symptoms  Severe asthma  Cough or phlegm | Odds ratio | Mobility  *High traffic density*  *Asthma symptoms WITHOUT cough or phlegm*:  OR=1.13; 95%CI=0.99, 1.28  *Cough or phlegm WITHOUT asthma symptoms*:  OR=1.14; 95%CI=0.92, 1.41  *Asthma symptoms WITH cough or Phlegm*:  OR=1.52; 95%CI=1.17, 1.96 | + |
|  | Santiago, B. M.  (2013) | To investigate the relationship of neighborhood social capital with dental pain in adolescents, adults and the elderly. | Paraiba;  Brazil | All individuals (15-74 years old)  *n=624* | Cross-sectional* | Prevalence of reported  dental pain in the last 6 months | Odds ratio | Safety  *Individual-level social capital*  *Bonding/Positive Interaction*: OR=0.88; 95%CI= 0.80, 0.91  *Neighborhood-level social capital*  *High social capital*: OR=0.48; 95%CI=0.27, 0.85 | + |
| **Morbidity related with mental illness** | Cau, B. M.  (2016) | To examine associations factors with mental health (poor self-rated health). | Maputo; Mozambique | Adults  (+ 40 years old)  *n=* *1768* | Cross-sectional* | SRH | Odds ratio | Sanitation  *Treated drinking water:*  OR= 0.49, p< 0.01 | + |
|  | Eibich, P.  (2016) | To study associations between neighborhood characteristics and mental health and well-being of younger (aged 20–35) and older (aged 60+) residents. | Berlin; Germany | Adults  (20-35/ +60 years old)  *n=2200* | Cohort | Life satisfaction  Health satisfaction  Self-assessed health  Physical health  Mental health  Morbidity index | Linear regression | Mobility  *Access to public transport*  *Life satisfaction*: β=0.67; ε=0.17  *Health satisfaction:* β=0.38; ε=0.19  *Self-assessed health:*  β= 1.82; ε=0.34  *Physical health*:  β= 1.86; ε=0.78  *Mental health*:  β= 2.04; ε=0.94  *Morbidity index*:  β= –0.30; ε=0.13 | + |
|  | Melis, G.  (2015) | To analyze the association of density, accessibility by public transport, accessibility to services, green and public spaces and mental disorders. | Turin;  Italy | Adults  (20-64 years old)  *n=547,263* | Longitudinal | Percentage of individuals who had any antidepressant prescription | Incidence Rate Ratios | Mobility  *Public transport*  *(high accessibility)*  *Men*: IRR= 0.93; SE=0.87, 0.98  *Women*: IRR=0.95; SE=0.92, 0.98 | + |
| **Morbidity related with obesity outcomes** | Lovasi, G. S. (2012) | To evaluate whether potentially attractive neighborhood features are associated with lower BMI, whether safety hazards are associated with higher BMI. | New York City;  USA | Adults  (+30 years old)  *n=13,102* | Cross-sectional | BMI  (kg/m^2^) | Interaction p-values | Mobility  *High walkability areas*  p=1.1; 95%CI=0.0, 2.8 | + |
|  | Lovasi, G. S. (2009) | To test whether the association between walkable environments and lower body mass index was stronger. | New York City;  USA | Adults  (+30 years old)  *n=13102* | Cross-sectional | BMI  (kg/m^2^) | Interaction p-values | Mobility  *Public transit use:*  p=-5.00 95%CI=-5.97, -4.02  *Subway access:*  p=-0.40; 95%CI=–0.52, –0.29  *Bus access*:  p=-0.07; 95%CI=–0.09, –0.05 | + |
|  | Mendes, L. L. (2013) | To evaluate variables within the built environment for their potential association with overweight and obesity. | Belo Horizonte; Brazil | Adults  (+18 years old)  *n=3404* | Cross-sectional | BMI  (kg/m^2^) | Prevalence ratios | Safety  *Homicide rate*:  PR=1.45; 95%CI=1.02, 2.05 | + |

Note: positive (+): a desirable improvement in the indicator was associated with an improvement of PH and negative (-): a desirable improvement in the indicator was associated with a deterioration of PH; † unclear information about population size; *”Study design” assigned by the authors

Table 4 Studies of built environment dimensions and analysed indicators and health outcomes without an association not statistically significant (for the defined statistical level)

| **Health outcome** | **Reference** | **Overall aim** | **City/Cities Country** | **Study population** | **Study design** | **Health outcome measure** | **Association measure** | **Dimension/ Indicator** | **Relation between indicator and PH** |
| --- | --- | --- | --- | --- | --- | --- | --- | --- | --- |
| **Overall mortality** | Ribeiro, A. I. (2016) | To evaluate the spatial distribution of old-age survival and its relationship with built environment and deprivation. | Porto;  Portugal | Elderly  (75–84 years old)† | Cohort* | Old-age survival | Odds ratio | Green Spaces  *NDVI*  *Men*  *-1 (least environmental deprived):* OR=1.02; 95%CI=0.90, 1.19  *+2 (most environmental deprived):* OR=1.04; 95%CI=0.92, 1.22  *Women*  *-1 (least environmental deprived):* OR=1.03; 95%CI=0.91, 1.21  *+2 (most environmental deprived):* OR=1.02; 95%CI=0.89, 1.17 | 0 |
|  |  |  |  |  |  |  |  | Mobility  *Walkability index*  *Men*  *1 (higher):* OR=0.90; 95%CI=0.72, 1.07  *10(lower):* OR=1.07; 95%CI= 0.91, 1.29  *Woman*  *1 (higher):* OR=0.86; 95%CI=0.69, 1.02  *10(lower):* OR=1.10; 95%CI= 0.95, 1.34 |  |
| **Morbidity related with mental illness** | Eibich, P.  (2016) | To study associations between neighborhood characteristics and mental health and well-being of younger (aged 20–35) and older (aged 60+) residents. | Berlin; Germany | Adults  (20-35/ +60 years old)  *n=2200* | Cohort | Life satisfaction  Health satisfaction  Self-assessed health  Physical health  Mental health  Morbidity index | Linear regression | Safety  *Crimes per capita (log)*  *Life satisfaction*: β=0.20; ε=0.39  *Health satisfaction:* β=0.35; ε=0.47  *Self-assessed health:*  β=2.12; ε=1.02  *Physical health*:  β=2.86; ε=1.86  *Mental health*:  β=1.19; ε=2.22  *Morbidity index*:  β=0.05; ε=0.3 | 0 |
| **Morbidity related with obesity outcomes** | Lange, D. (2011) | To evaluate the influence of neighborhood characteristics on adolescent BMI. | Kiel;  Germany | Adolescents (13-15 years old)  *n=3440* | Cross-sectional | BMI  (kg/m^2^) | Linear multilevel associations | Mobility  *Traffic density*  r=0.00; p=0.05 | 0 |
|  |  |  |  |  |  |  |  | Safety  *Crime rate*  r=0.01; p=0.05 |  |
|  |  |  |  |  |  |  |  | Green spaces  *Nr of parks*  r=0.07; p=0.05 |  |
|  | Lovasi, G. S. (2012) | To evaluate whether potentially attractive neighborhood features are associated with lower BMI, whether safety hazards are associated with higher BMI. | New York City;  USA | Adults  (+30 years old)  *n=13,102* | Cross-sectional | BMI  (kg/m^2^) | Interaction p-values | Safety Homicide prevalence  p=−0.06; 95%CI=−0.37, 0.25 | 0 |
|  | Lovasi, G. S. (2011) | To examine which built environment characteristics correlates of physical activity and anthropometry among preschool children. | New York City;  USA | Children  (2–5 years old)  *n=428* | Cross-sectional | BMI z-score | Regression models | Mobility  *Walkability*  *Population density*: α=−0.08; 95%CI=−0.38, 0.22  *Subway stop density*: α=−0.16; 95%CI=−0.33, 0.01  *Bus stop density*: α=0.10; 95%CI=−0.09, 0.30 | 0 |
|  |  |  |  |  |  |  |  | Safety  *Crime rate*  *Homicide rate*: α=0.04; 95%CI=−0.19, 0.26 |  |
|  | Mendes, L. L. (2013) | To evaluate variables within the built environment for their potential association with overweight and obesity. | Belo Horizonte; Brazil | Adults  (+18 years old)  *n=3404* | Cross-sectional | BMI  (kg/m^2^) | Prevalence ratios | Mobility  *Population density*  *2nd /3rd/ 4th quartile*: PR=0.85; 95%CI= 0.74, 0.98 | 0 |
|  |  |  |  |  |  |  |  | Green spaces  *Parks/public squares/places for practicing physical activity*  *Yes:* PR=0.99; 95%CI=0.72, 1.37 |  |

† unclear information about population size; *” Study design” assigned by the authors

Table 5 Studies of natural environment dimensions and analysed indicators and health outcomes with evidence of association

| **Health outcome** | **Reference** | **Overall aim** | **City/Cities Country** | **Study population** | **Study design** | **Health outcome measure** | **Association measure** | **Dimension/ Indicator** | **Relation between indicator and PH** |
| --- | --- | --- | --- | --- | --- | --- | --- | --- | --- |
| **Overall mortality** | Hu, W.  (2008) | To investigate the effect of temperature and air pollutants on total mortality in summers. | Sydney; Australia | All individuals† | Ecological* | Number of daily mortality | Relative risk | Air quality  *SO_2_*:  RR=22; 95%CI=6.4, 40.5 | **+** |
|  | Kioumourtzoglou, M. A.  (2016) | To estimate effects between long-term PM_2.5_ exposures and mortality. | 207 cities,  USA | Elderly  (65 years old)  *n=>35 million Medicare enrollees* | Cohort | Number of deaths | Hazard ratios | Air quality  *PM_2.5_*  HR= 1.19; 95%CI=1.11, 1.28 *per 10 µg/m^3^ increase in the annual PM2.5concentrations* | **+** |
|  | Li, H.  (2015) | To estimate the effects of outdoor air pollution on daily coronary heart disease mortality. | Beijing, Shanghai, Guangzhou, Hong Kong, Shenyang, Tangshan, Taiyuan, Xi'na; China | All individuals  *n=48.3 million* | Cross-sectional* | Daily coronary heart disease mortality | Interquartile range | Air quality  *PM_10_*: IQR=0.36; 95%CI=0.12, 0.61  *SO_2_*: IQR=0.86; 95%CI=0.30, 1.41  *NO_2_*: IQR=1.30; 95%CI=0.45, 2.14 | **+** |
|  | Luo, K.  (2017) | To assess the effect modification of cardiovascular mortality by air pollutants. | Beijing, Nanjing, Chengdu; China | Elderly  (65 years old)  *n=290 593 deaths* | Cross-sectional* | Daily mortality | Percentage increase mortality per 1ºC | Air quality  *PM_10_*  *Time model (6 degree of freedom (DF)):* α=1.52; 95%CI=0.85, 2.11  *Temperature model (6 DF):* α=1.31; 95%CI=0.72, 1.92 | **+** |
|  | Neuberger, M. (2013) | To assess the effect of daily pollution on the relationship with daily mortality. | Vienna, Graz, Linz; Austria | All individuals† | Case-crossover | All causes daily mortality | Percentage increase risk | Air quality  *Vienna (Lag 0–14 days)*  *PM_2.5_*: IR=2.6; 95%CI=1.1, 4.1  *PM_10_:* IR=1.2; 95%CI=0.4, 2.1  *TSP (total suspended particles)*: IR=0.8; 95%CI=0.0, 1.6  *NO_2_*: IR=2.9; 95%CI=1.6, 4.1  *Graz (Lag 0–14 days)*  *PM_10_:* IR=1.6; 95%CI=1.1, 2.0  *TSP*: IR=1.2; 95%CI=0.9, 1.5  *NO_2_*: IR=2.6; 95%CI=2.0, 3.1  *Linz (Lag 0–14 days)*  *PM_2.5_*: IR=1.2; 95%CI=0.7, 1.7  *PM_10_:* IR=0.6; 95%CI=0.4, 0.9  *NO_2_*: IR=1.5; 95%CI=0.9, 2.2 | **+** |
|  | Willers, S. M. (2016) | To investigate the extent of neighborhood differences in mortality risk due to heat and air pollution in a city with a temperate maritime climate. | Rotterdam; Netherlands | All individuals (<45 years old)  *n=73,178 deaths* | Case-crossover | Natural-cause mortality cases | Percentage increase risk | Air quality  *PM_10_*  *Lag time 0:* IR=1.7; 95%CI= 0.8, 2.6  *Lag time 1:* IR=1.6; 95%CI= 0.6, 2.5  *Lag time 2:* IR=1.2; 95%CI= 0.2, 2.1 | **+** |
|  | Wong, C. M. (2008) | To examine whether people residing in socially deprived communities are at higher mortality risk from ambient air pollution. | Hong Kong; China | All individuals† | Ecological* | All registered deaths | Excess risk | Air quality  *NO_2_*: ER=0.16; 95%CI=-0.07, 0.39  *SO_2_*: ER=0.45; 95%CI=0.03, 0.87  *PM_10_*: ER= 0.04; 95%CI= -0.15, 0.22  *O_3_:* ER=0.05; 95%CI= -0.16, 0.25 | **+** |
| **Morbidity related with birth outcomes** | Huang, J. V. (2017) | To examine sex-specific associations of particulate PM_10_, nitric oxide, sulfur dioxide, and nitrogen dioxide in different growth phases with clinically assessed pubertal stage. | Hong Kong; China | Children  (9–12 years old)  *n= 8327* | Cohort | Highest Tanner stage | Mean difference | Air quality  *PM_10_* *(in utero)*  Boys: D_f_=0.01; 95%CI=-0.01, 0.03  Girls: D_f_=0.05; 95%CI=-0.08, -0.02  *SO_2_*:  Boys: D_f_=-0.03; 95%CI=-0.05, -0.01  Girls: D_f_=-0.03; 95%CI=-3.8e-3, 0.06  *NO_2_*:  Boys: D_f_=0.03; 95%CI=-0.04, -0.02  Girls: D_f_=-0.01; 95%CI=-0.03,0.01 | **+** |
| **Morbidity related with chronic diseases outcomes** | Dzhambov, A. M.  (2016) | To explore the overall association of prevalence of Type 2 diabetes mellitus (T2DM) with exposures to road traffic, noise and air pollution. | Plovdiv; Bulgaria | Adults  (+18 years old)  *n=* *513* | Cross-sectional | T2DM self-reported doctor diagnosis | Odds Ratio | Noise  *L_den_*:  OR=4.49; 95%CI=1.38, 14.68 | **+** |
|  |  |  |  |  |  |  |  | Air quality  *PM_2.5_*: OR=1.32; 95% CI=0.28,6.24  *B[a]pyrene*: OR=1.76; 95% CI=0.52, 5.98 |  |
|  | Lemke, L. D.  (2014) | To investigate ambient air quality across the border between and its association with acute asthma events. | Detroit, Michigan; USA  Windsor, Ontario; Canada | All individuals (+5 years old) † | Cross-sectional* | Asthma prevalence | Pearson correlation coefficients | Air quality  *VOCs (volatile organic compounds)*  Þ=0.51; 95%CI=0.14, 0.75 *BTEX (benzene, toluene, ethylbenzene and xylene)*  Þ=0.53; 95%CI=0.17, 0.77 *NO_2_*  Þ=0.63; 95%CI=0.08, 0.88 *PM_10_*  Þ=0.61; 95%CI=0.06, 0.88 | **+** |
|  | Liu, C.  (2014) | To investigate associations between long-term traffic-related air pollution and long-term noise exposure with blood pressure (BP) in children. | Munich, Wesel; Germany | Children  (10 years old)  *n=2368* | Cohort | Resting systolic and diastolic BP | Spearman’s rank correlation coefficient | Noise  *L_den_:* β=1.00; p=0.05  *L_dnight_:* β =0.92; p<0.001 | **+** |
|  | Modig, L.  (2009) | To investigate the relationship between the cumulative incidence of asthma and onset of asthma among adults and vehicle exhaust concentrations at home. | Gothenburg, Uppsala, Umeå; Sweden | Adults  (20–44 years old)  *n=10,800* | Cohort | Cases and noncases of asthma | Odds Ratio | Air quality  *NO_2_*  *Onset asthma*: OR=1.46; 95%CI=1.07, 1.99  *Incident asthma:* OR=1.54; 95%CI=1.00, 2.36 | **+** |
|  | Patel, M. M. (2010) | To examine associations of daily ambient black carbon concentrations with daily respiratory symptoms among asthmatic and non-asthmatic adolescents. | New York City;  USA | Adolescents (13–20 years old)  *n=249* | Longitudinal | Self-reported asthma | Odds Ratio | Air quality  *NO_2_*  *Asthma:* OR=1.13; 95%CI=0.94, 1.36  *No Asthma:* OR=0.90; 95%CI=0.79, 1.02 | **+** |
|  | Sun, G.  (2017) | To assess potential carcinogenic and non-carcinogenic health effects on children and adults due to exposure to street dust in China. | Zhuzhou; China | All individuals† | Cohort* | Lifetime average daily dose (mg/kg^−1^/day^−1^) | Hazard quotient | Air quality  *As*  *Adults*: HQ= 4.47×10^−1^  *Children*: HQ= 3.18×10  *Cr*  *Adults*: HQ= 4.45×10^−2^  *Children*: HQ= 3.15×10^−1^ | **+** |
|  | Yang, B. Y. (2017) | To evaluate the effects of long-term exposure to ambient air pollution PM_10_, sulfur dioxide, nitrogen dioxide, ozone on prehypertension. | Shenyang, Anshan, Jinzhou;  China | Adults  (18-74 years old)  *n=24,845* | Cross-sectional* | BP values | Odds Ratio | Air quality  *PM_10_*: OR=1.17; 95%CI=1.09, 1.25  *SO_2_*: OR=1.11; 95%CI=1.00, 1.25  *NO_2_:* OR=1.18; 95%CI=1.05, 1.32  *O_3_:* OR=1.13; 95%CI=0.99, 1.28 | **+** |
| **Morbidity related with mental illness** | Grelat, N.  (2016) | To quantify the annoyance caused by noise in children and to assess the relationship between these children's noise annoyance level and factors in the surrounding urban area. | Besançon; France | Children  (7–11 years old)  *n=517* | Cross-sectional* | Children’s Noise Annoyance | Odds Ratio | Noise  *Ambient noise (dBA):*  OR=0.77; 95%CI=0.51, 1.16 | **+** |
|  | Habib, R. R. (2013) | To evaluate the association between women’s SRH and socioeconomic indicators. | Bebnine, Lebanon | Adult  (+14 years old)  *n=2223* | Cross-sectional | Self-reported health status | Odds Ratio | Water quality  *Satisfaction with quality of drinking water*  *Fair/Good*: OR=1.16; 95%CI=0.98, 1.62  *Poor/ Good*: OR=1.45; 95%CI=1.13, 1.88 | **+** |
|  | Ristovska, G. (2009) | To identify noise exposure indicators during day and night and to evaluate if there is an association between those and annoyance. | Skopje; Macedonia | Adult  (18- 65 years old)  *n=510* | Cross-sectional* | Annoyance with noise over the last 12 months | Spearman’s rank order | Noise  *L_day_ dB(A)*:  r_s_= 0.45; p<0.05  *L_night_ dB(A)*:  r_s_= -0.125; p<0.05 | **+** |

Note: positive (+): a desirable improvement in the indicator was associated with an improvement of PH and negative (-): a desirable improvement in the indicator was associated with a deterioration of PH; † unclear information about population size; *”Study design” assigned by the authors

Table 6 Studies of natural environment dimensions and analysed indicators and health outcomes without an association not statistically significant (for the defined statistical level)

| **Health outcome** | **Reference** | **Overall aim** | **City/Cities Country** | **Study population** | **Study design** | **Health outcome measure** | **Association measure** | **Dimension/ Indicator** | **Relation between indicator and PH** |
| --- | --- | --- | --- | --- | --- | --- | --- | --- | --- |
| **Overall mortality** | Luo, K.  (2017) | To assess the effect modification of cardiovascular mortality by air pollutants. | Beijing, Nanjing, Chengdu; China | Elderly  (65 years old)  *n=290 593 deaths* | Cross-sectional* | Daily mortality | Percentage increase mortality per 1ºC | Air quality  *NO_2_*  *Time model (6 DF):* α=1.35; 95%CI=0.77,1.95  *Temperature model (6 DF):* α=1.16; 95%CI=0.49,1.85  *SO_2_*  *Time model (6 DF):* α=1.36; 95%CI= 0.77,1.94  *Temperature model (6 DF):* α=1.16; 95%CI=0.45,1.88 | 0 |
|  | Pasetto, R.  (2013) | To assess all-cause mortality of the population living in a polluted area, built over an area with chlorinated organic compounds. | Ferrara;  Italy | All individuals  *n=3475* | Cohort | Mortality incidence | Standardized mortality ratio | Water quality  *Chlorinated organic compounds*  SMR=81;90%CI=73, 89 | 0 |
| **Morbidity related with chronic diseases** | Liu, C.  (2014) | To investigate associations between long-term traffic-related air pollution and long-term noise exposure with blood pressure (BP) in children. | Munich, Wesel; Germany | Children  (10 years old)  *n=2368* | Cohort | Resting systolic and diastolic BP | Spearman’s rank correlation coefficient | Air quality  *NO_2_:* β =0.11; 95%CI=-0.45, 0.67  *PM_10_*: β =0.25; 95%CI=-0.71, 1.21  *PM_2.5_*: β =1.01; 95%CI=-0.90, 2.92 | 0 |
|  | Sun, G.  (2017) | To assess potential carcinogenic and non-carcinogenic health effects on children and adults due to exposure to street dust in China. | Zhuzhou; China | All individuals† | Cohort* | Lifetime average daily dose (mg/kg^−1^/day^−1^) | Hazard quotient | Air quality  *Pb*  *Adults*: HQ= 4.36×10^−1^  *Children*: HQ=3.17×10  *Cd*  *Adults*: HQ=7.40×10^−2^  *Children*: HQ=5.22×10^−1^  *Hg*  *Adults*: HQ=1.09×10^−1^  *Children*: HQ=1.79×10^−1^  *Sb*  *Adults*: HQ=5.14×10^−2^  *Children*: HQ=3.67×10^−1^ | 0 |

† unclear information about population size; *” Study design” assigned by the authors

Table 7 Studies of healthcare dimensions and analysed indicators and health outcomes with evidence of association

| **Health outcome** | **Reference** | **Overall aim** | **City/Cities Country** | **Study population** | **Study design** | **Health outcome measure** | **Association measure** | **Dimension/ Indicator** | **Relation between indicator and PH** |
| --- | --- | --- | --- | --- | --- | --- | --- | --- | --- |
| **Overall Mortality** | Cheng, E. R. (2012) | To describe the association premature mortality among counties with broadly differing levels of income. | 3,139 USA counties | All individuals (to 75 years old) † | Cross-sectional* | All-cause, age-adjusted mortality rate per 100,000 population | β coefficient | Hospital care  *Primary care providers per 1,000*  β=0.01; SE=0.01 | **+** |
|  | James, W. L. (2012) | To understand if overall and race-specific mortality rates are combined with local health infrastructure data, income inequality and racial segregation. | 48 USA states | All individuals† | Ecological | Race-specific age/sex adjusted mortality rates per 100,000 | Moran’s I coefficient | Hospital care  *Health infrastructures*  *Whites*: I=-2.031; p<0.01  *Black*: I=5.089; p>0.05 | **+** |
|  | Santos, S. L.  (2016) | To identify factors associated with infant mortality by a hierarchical model based on socioeconomic determinants like maternal education and maternal occupation. | Teresina; Brazil | Newborns (Mother age: +10 years old)  *n=13,882* | Cohort | Number of love births and numbers of death births | Odds ratio | Hospital care  *Health infrastructures*  OR= 1.28; 95%CI=1.11, 1.51 | **+** |

Note: positive (+): a desirable improvement in the indicator was associated with an improvement of PH and negative (-): a desirable improvement in the indicator was associated with a deterioration of PH; † unclear information about population size; *”Study design” assigned by the authors

Table 8 Studies of healthcare dimensions and analysed indicators and health outcomes without an association not statistically significant (for the defined statistical level)

| **Health outcome** | **Reference** | **Overall aim** | **City/Cities Country** | **Study population** | **Study design** | **Health outcome measure** | **Association measure** | **Dimension/ Indicator** | **Relation between indicator and PH** |
| --- | --- | --- | --- | --- | --- | --- | --- | --- | --- |
| **Morbidity related with chronic diseases** | Bastos, L. N. V.  (2018) | To determine the incidence and association with environmental health indicators of cases of childhood and adolescent cancer. | Pernambuco; Brazil | Adolescents (<20 years old)  *n=* *1261* | Cross-sectional | Average Age-adjusted Incidence Rate of Cancer | Moran index | Hospital care  *Basic health facilities per capita*  MI= 0.011225; p= 0.715 | 0 |
|  | Ferreira-Junior, O. M.  (2015) | To assess prevalence of dental pain and associated contextual factors. | 27 capital cities  Brazil | Children  (5 years old)  *n=7280* | Cross-sectional | Dental pain in the last 6 months | Prevalence rate | Oral care  *Dental visits*  PR=1.14; 95%CI=1.20, 1.65 | 0 |

† unclear information about population size; *” Study design” assigned by the authors

Table 9 Studies of health behaviors dimensions and analysed indicators and health outcomes with evidence of association

| **Health outcome** | **Reference** | **Overall aim** | **City/Cities Country** | **Study population** | **Study design** | **Health outcome measure** | **Association measure** | **Dimension/ Indicator** | **Relation between indicator and PH** |
| --- | --- | --- | --- | --- | --- | --- | --- | --- | --- |
| **Overall Mortality** | Cheng, E. R. (2012) | To describe the association premature mortality among counties with broadly differing levels of income. | 3,139 USA counties | All individuals (to 75 years old) † | Cross-sectional* | All-cause, age-adjusted mortality rate per 100,000 population | β coefficient | Smoking  *% residents who smoke*  β=0.05; SE= 0.01 | **+** |
|  | Maniecka-Bryla, I.  (2013) | To identify risk factors influencing the phenomenon of all cause’s premature deaths. | Łódź;  Poland | Adults  (18-64 years old)  *n=4000* | Cohort* | Number of premature deaths | Hazard ratio | Alcohol consumption  *Drinking style*  *drinks in moderation:* HR=0.486; 95%CI= 0.271, 0.869  *drinks excessively:* HR=1.444; 95%CI=1.191, 2.031 | **+** |
|  |  |  |  |  |  |  |  | Smoking  *Smoking nicotine*  *used to smoke:* HR= 1.125; 95%=CI= 0.515, 2.456  *smokes:* HR= 2.782; 95%=CI= 1.581, 4.891 |  |
|  | Smigielski, J. (2013) | To identify the relationships on the mortality of men of subjective health assessment, nutritional habits, alcohol consumption and tobacco smoking. | Łódź;  Poland | Adults  (18-64 years old)  *n=1004* | Cross-sectional* | Recorded deaths | Survival probability | Alcohol consumption  *Beer drinking:* C=1.99; p<0.05  *Wine drinking:* C=2.47; p<0.01 | + |
|  |  |  |  |  |  |  |  | Smoking  *History of tobacco smoking:* C=1,92; p<0.05 |  |
|  |  |  |  |  |  |  |  | Nutrition  *Additional use of table salt at meals:* C=1.80; p<0.05 |  |
| **Morbidity related with chronic diseases** | Artaud, F.  (2013) | To examine the individual and combined associations of unhealthy behaviors with hazard of disability among older French adults. | Bordeaux, Dijon, Montpellier; France | Elderly  (+65 years old)  *n=* *3982* | Cohort | Hierarchical indicator of disability (no, light, moderate, severe) | Hazard ratio | Nutrition  *Consumption of fruit and vegetables (<4 times a week):*  HR=1.25, 95%CI=1.10, 1.41 | + |
|  |  |  |  |  |  |  |  | Smoking  *Current smoker or short-term ex-smoker* vs  *never smoker or long-term ex-smoker*  HR=1.31, 95%CI=1.07, 1.60 |  |
|  |  |  |  |  |  |  |  | Physical activity  *Low or intermediate* vs  *High*  HR=1.72, 95%CI=1.48, 2.00 |  |
|  | Cabral, D. M. (2014) | To determine the prevalence of chronic pain and to identify sociodemographic, psychosocial, and occupational factors associated. | São Paulo;  Brazil | Adults  (+15 years old)  *n=* *1,108* | Cross-sectional | Chronic Pain Grade (CPG) | Prevalence ratio | Smoking  *Current smoker*  PR=1.22; 95%CI=1.02, 1.47 | + |
|  |  |  |  |  |  |  |  | Physical activity  *Intense or heavy activity* PR=1.40; 95%CI=1.06, 1.86 |  |
|  | de Carvalho Cremm, E. (2012) | To investigate the individual and family determinants of being overweight among children living in an urban area. | Santos;  Brazil | Children (<10 years old)  *n=* *531* | Cross-sectional | BMI-for-age  (kg/m^2^) | Odds ratio | Physical activity  *Transportation (walks/bikes)*  OR=1.70; p=0.05 | + |
|  | Eisele, M.  (2015) | To investigate factors influencing the course of health-related quality of life in older primary care patients and to derive non-pharmacological recommendations for improving their quality of life. | Bonn, Düsseldorf, Hamburg, Leipzig, Mannheim, Munich; Germany | Elderly  (≥78 years)  *n=1968* | Prospective longitudinal observational | Health-related  quality of life (HRQoL) | β coefficient | Physical activity  *Activity level*  β=1.011, 95%CI=0.127, 1.895 | + |
|  | Pizzo, G. (2010) | To investigate the prevalence of caries in children and assessed the relationship between socio-behavioral determinants and caries. | Palermo; Italy | Children  (5 and 12 years old)  *n=511* | Cross-sectional* | Dmf-t index | Odds ratio | Nutrition  *Frequency of snack consumption*  *More than once a day* vs *Once a day:* OR= 1.92, 95%CI=1.04, 3.54 | + |
|  | Sanderson, M. (2015) | To determine whether physical activity and adult weight change, are linked to breast cancer risk. | Nashville; USA | Adult women  (25 to 75 years old)  *n=2614 incident breast cancer cases*  *and 2306 controls* | Case-control | Population-based case-control | Odds ratio | Physical activity  *Hours per day postmenopausal*  *White*  *0.1–1.4:* OR= 0.8, 95%CI=0.6, 1.0  *1.5–3.6:* OR=1.0, 95%CI=0.8, 1.2)  *≥3.7:* OR= 0.8, 95%CI=0.6, 1.0  *Black*  *0.1–1.4:* OR= 0.7, 95%CI=0.5, 1.2  *1.5–3.6:* OR= 0.7, 95%CI=0.4, 1.2  *≥3.7:* OR= 0.7, 95%CI=0.4, 1.1 | + |
|  | Schulz, A. J. (2008) | To provide evidence of an effect linking socioeconomic indicators to cardiovascular disease (CVD) risk factors. | Detroit, Michigan; USA | Adults  (≥25 years old)  *n=919* | Cross-sectional* | Depressive symptoms, smoking status, physical activity,  body mass index and waist circumference | β coeficient | Smoking  *Current smoker*  *Body mass index:* β=-3.23, p<0.001  *Waist circumference*:  β =-4.40, p<0.001 | + |
| **Morbidity related with mental illness** | Cau, B. M.  (2016) | To examine associations factors with mental health (poor self-rated health). | Maputo; Mozambique | Adults  (+ 40 years old)  *n=* *1768* | Cross-sectional* | SRH | Odds ratio | Physical activity  *Intensive activity*  OR=0.60; p<0.05 | + |
|  | Christiani, Y. (2015) | To examine unequally distribution of depression among women in Indonesia's major cities and investigate the factors contributed to the inequality. | Jakarta, Surabaya, Medan, Bandung; Indonesia | Adult  (19-65 years old)  *n=1117* | Cross-sectional* | Prevalence of depression | Concentration index | Smoking  *Current smoker*  CI= -0.151, p<0.05 | + |
|  | Franca, V. F. (2016) | To assess the association between unhealthy dietary habits and cognition in older adults. | Florianopolis  Brazil | Adults  (+60 years old)  *n=1197* | Cross-sectional | Mini-Mental State Examination (MMSE) | β coefficient | Nutrition  *Unhealthy dietary habits*  *Women*  *Fruits and Vegetables:* β=-1.004;  95%CI= -1.376, -0.631  *Fish:* β=0.073;  95%CI= -0.312, 0.458  *Red meat with fat:*  β=-0.171; 95%CI= -0.873, 1.216  *Chicken skin:* β=0.290; 95%CI= -0.756, 1.336  *Men*  *Fruits and Vegetables:* β=0.298;  95%CI= -0.393, 0.968  *Fish:* β=-0.441;  95%CI= -1.059, 0.177  *Red meat with fat:* β=0.505;  95%CI= -0.415, 1.424  *Chicken skin:* β=0.008, 95%CI= -1.103, 1.120 | + |
|  | Ribeiro Dos Santos, E. (2016) | To estimate the prevalence of depression associated with socio-demographic and economic characteristics, smoking habits, alcohol use, and physical morbidities. | Coari and Tefé;  Brazil | Adults  (+20 years old)  *n=34,838* | Cross-sectional | Score of ≥10 on the Patient Health Questionnaire-9 (PHQ-9) | Prevalence ratio | Smoking  *Current smoker*  PR=1.12; 95%CI=0.82, 1.52 | + |
|  |  |  |  |  |  |  |  | Alcohol consumption  *Risky alcohol use* PR=1.30; 95%CI=0.99, 1.77 |  |

Note: positive (+): a desirable improvement in the indicator was associated with an improvement of PH and negative (-): a desirable improvement in the indicator was associated with a deterioration of PH; † unclear information about population size; *”Study design” assigned by the authors

Table 10 Studies of health behaviors dimensions and analysed indicators and health outcomes without an association not statistically significant (for the defined statistical level)

| **Health outcome** | **Reference** | **Overall aim** | **City/Cities Country** | **Study population** | **Study design** | **Health outcome measure** | **Association measure** | **Dimension/ Indicator** | **Relation between indicator and PH** |
| --- | --- | --- | --- | --- | --- | --- | --- | --- | --- |
| **Overall Mortality** | Smigielski, J. (2013) | To identify the relationships on the mortality of men of subjective health assessment, nutritional habits, alcohol consumption and tobacco smoking. | Łódź;  Poland | Adults  (18-64 years old)  *n=1004* | Cross-sectional* | Recorded deaths | Survival probability | Physical activity  *Small or not at all vs. moderates vs. high*): C=0.064; p<0.05 | 0 |
| **Morbidity related with chronic diseases** | Artaud, F.  (2013) | To examine the individual and combined associations of unhealthy behaviors with hazard of disability among older French adults. | Bordeaux, Dijon, Montpellier; France | Elderly  (+65 years old)  *n=3982* | Cohort | Hierarchical indicator of disability (no, light, moderate, severe) | Hazard ratio | Alcohol consumption  *Never, former, or*  *heavy drinker* vs *light to*  *moderate drinker*  HR=1.00, 95%CI=0.92, 1.09 | 0 |
|  | Cabral, D. M. (2014) | To determine the prevalence of chronic pain and to identify sociodemographic, psychosocial, and occupational factors associated. | São Paulo;  Brazil | Adults  (+15 years old)  *n=* *1,108* | Cross-sectional | Chronic Pain Grade (CPG) | Prevalence ratio | Alcohol consumption  *Hazardous alcohol use/dependence symptoms/harmful alcohol use*  PR=0.84; 95%CI=0.61, 1.16 | 0 |
|  | Fatema, K.  (2013) | To evaluate the proportion of urban adults suffering from chronic kidney disease as well as to have a preliminary idea about the determinants of this disorder. | Dhaka; Bangladesh | Adults  (+18 years old)  *n=634* | Cohort | “Likely CKD” according to Kidney Disease: Improving Global Outcomes | Odds ratio | Smoking  *Current smoker*  OR=1.7; 95%CI=0.9, 3.08 | 0 |
|  | Schulz, A. J. (2008) | To provide evidence of an effect linking socioeconomic indicators to cardiovascular disease (CVD) risk factors. | Detroit, Michigan; USA | Adults  (≥25 years old)  *n=919* | Cross-sectional* | Depressive symptoms, smoking status, physical activity,  body mass index and waist circumference | β coeficient | Smoking  Physical activity  β =-0.65, p<0.001 | 0 |
| **Morbidity related with mental illness** | Franca, V. F. (2016) | To assess the association between unhealthy dietary habits and cognition in older adults. | Florianopolis  Brazil | Adults  (+60 years old)  *n=1197* | Cross-sectional | Mini-Mental State Examination (MMSE) | β coefficient | Alcohol consumption  *No alcohol intake*  p< 0.001 | 0 |
|  |  |  |  |  |  |  |  | Smoking  *Non-smoker*  p=0.006 |  |
| **Morbidity related with obesity outcomes** | Albaladejo, R.  (2014) | To assess whether the relationship between childhood obesity is explained by family socioeconomic position and risk behaviors. | Madrid; Spain | Children  (6–15 years old)  *n=* *727* | Cross-sectional* | BMI  (kg/m^2^) | Odds ratio | Physical activity  *≤1.01 Sport facilities*  *per 1000 population*  OR=1.10; 95%CI=0.53, 2.25 | 0 |

† unclear information about population size; *”Study design” assigned by the authors
