# Supplementary material for: Environmental determinants of population health in urban settings. A systematic review
Source: BMC Public Health. 2020 Jun 3;20:853. doi: 10.1186/s12889-020-08905-0 (PMC7271472; doi:10.1186/s12889-020-08905-0)
Supplement: Supplementary file 2 — Additional file 2. [file 12889_2020_8905_MOESM2_ESM.docx]

Table 1 Risk of bias assessment (source: Rojas-Rueda D, 2019)

|  | **Bias due to exposure assessment** | **Bias due to confounding** | **Bias due to selection of participants** | **Bias due to health outcome assessment** | **Bias due to not blinded outcome assessment** | **Total risk of bias** |
| --- | --- | --- | --- | --- | --- | --- |
| **Low** | A clear description of the exposure assessment and exposure unit; based on measurements or modeling. | All-important confounders are considered either through matching or, restriction or in the analysis. (e.g., age, gender, etc.) | Participants randomly sampled from a known population, AND  response rate higher than 60%,  AND  attrition rate less than 20% in follow-up studies. | The health outcome of interest is objectively measured OR taken from medical records OR taken from questionnaire or interview using a known scale or validated assessment method | The health outcome of interest is assessed blind for exposure information in cohort and cross-sectional studies or exposure is assessed blind for being a case in case-control studies | At least 4 at low risk of bias. One “high” or “unclear” out of five is allowed |
| **High** | Not clear description of the exposure assessment or exposure unit OR/AND  performed by unqualified staff | Only 1 or no confounder is taken into account;  OR subjects in exposed and unexposed groups differ for one or more important confounders and there is no adjustment in the analysis | No random sampling OR  response rate less than 60%  OR  attrition rate higher than 20%. | The health outcome of interest is self-reported and not assessed using a known scale or validated assessment method. | The health outcome and/or exposure assessment is not blinded. | Any other |
| **Unclear** | Not enough information is available to judge the above | Less then all to > 1 important confounder taken into account, OR Insufficient information to decide on one of the above. | No information to judge the above. | Not enough information reported to assess the above. | Not enough information reported to assess the above. |  |
| **Not apply** | N/A | N/A | N/A | N/A | N/A |  |

Table 2 Risk of bias assessment for all reviewed studies

| **Reference** | **Bias due to exposure assessment** | **Bias due to confounding** | **Bias due to selection of participants** | **Bias due to health outcome assessment** | **Bias due to not blinded outcome assessment** | **Total risk of bias** |
| --- | --- | --- | --- | --- | --- | --- |
| Aguilar-Palacio, I. (2012) | High | Low | Low | High | Unclear | **High** |
| Akarolo-Anthony, S.N. (2014) | Low | Low | Low | Low | High | **Low** |
| Albaladejo, R. (2014) | Low | Low | Unclear | High | High | **High** |
| Ali, M.K. (2016) | Low | Low | Low | Low | High | **Low** |
| Artaud, F. (2013) | Low | Low | High | High | Unclear | **High** |
| Banerjee, D. (2010) | High | Low | Low | High | High | **High** |
| Bastos, L.N.V. (2018) | Low | Low | Unclear | Low | High | **High** |
| Belon, A.P. (2012) | High | High | Unclear | High | N/A | **High** |
| Borrell, C. (2014) | Low | Low | Unclear | Low | High | **High** |
| Buot, M.L. (2014) | Low | Low | Low | Low | N/A | **Low** |
| Cabral, D.M. (2014) | Low | Low | Low | High | Low | **Low** |
| Cau, B.M. (2016). | Low | Low | Low | High | Unclear | **High** |
| Ceccon, R.F. (2014) | Unclear | Unclear | N/A | Low | N/A | **High** |
| Cheng, E.R. (2012) | Low | Unclear | N/A | Low | N/A | **High** |
| Christiani, Y. (2015) | Low | High | High | Low | High | **High** |
| Cordoba-Dona, J.A. (2012) | Low | High | Unclear | Low | High | **High** |
| de Carvalho Cremm, E. (2012) | Low | Low | Low | Low | High | **Low** |
| de Sousa Gdos, S. (2014) | Low | Unclear | High | Low | N/A | **High** |
| de Souza, O.F. (2012) | Low | Unclear | Low | Low | Unclear | **High** |
| Duarte-Salles, T. (2011) | Low | High | Unclear | Low | High | **High** |
| Dzhambov, A.M. (2016) | Low | Low | Low | Low | High | **Low** |
| Eibich, P. (2016) | Low | Low | Low | Low | High | **Low** |
| Eisele, M. (2015) | Low | Low | Unclear | High | High | **High** |
| Enroth, L. (2013) | Low | High | High | High | High | **High** |
| Faresjo, T. (2010) | Low | Unclear | High | High | High | **High** |
| Fatema, K. (2013) | Low | Low | High | High | High | **High** |
| Ferreira-Junior, O.M. (2015) | Low | Low | Low | Unclear | Unclear | **High** |
| Fleischer, N.L. (2008) | Low | Low | High | Low | High | **High** |
| Franca, M.H. (2017) | Low | Low | Low | High | High | **High** |
| Franca, V.F. (2016) | Low | High | Unclear | High | Unclear | **High** |
| Garcia-Subirats, I. (2011) | Low | High | Low | Low | High | **High** |
| Goulart, M.D. (2016) | Low | Low | Low | Low | High | **Low** |
| Grazuleviciene, R. (2015) | Low | Low | Low | Low | High | **Low** |
| Grelat, N. (2016) | Low | Low | Low | High | High | **High** |
| Gronlund, C.J. (2015) | Low | Low | Low | Low | High | **Low** |
| Habermann, M. (2012) | Low | Unclear | N/A | Low | N/A | **High** |
| Habib, R.R. (2013) | Low | Low | Low | High | High | **High** |
| Harlan, S.L. (2013) | High | Low | N/A | Low | N/A | **High** |
| Hayward, I. (2012) | Low | Low | Low | Low | N/A | **Low** |
| Hu, W. (2008) | Low | Unclear | N/A | Low | High | **High** |
| Huang, J.V. (2017) | Low | Low | Unclear | High | High | **High** |
| James, W.L. (2012) | Low | Low | High | Low | N/A | **High** |
| Kioumourtzoglou, M.A. (2016) | Low | Unclear | N/A | Low | Unclear | **High** |
| Lacerda, J.T. (2008) | Low | Low | Low | High | High | **High** |
| Lange, D. (2011) | Low | High | Unclear | Low | High | **High** |
| Lee, J. (2014) | Low | Low | Unclear | Low | N/A | **High** |
| Lemke, L.D. (2014) | Low | Unclear | Unclear | Unclear | High | **High** |
| Li, H. (2015) | Low | Unclear | N/A | Low | Low | **High** |
| Liu, C. (2014) | Low | Low | Low | Low | High | **Low** |
| Lopes, E.M. (2015) | Low | Unclear | Low | Low | High | **High** |
| Lovasi, G.S. (2012) | High | Low | Unclear | Unclear | High | **High** |
| Lovasi, G.S. (2011) | Low | High | Unclear | Low | High | **High** |
| Lovasi, G.S. (2009) | High | Low | Unclear | Low | High | **High** |
| Luo, K. (2017) | High | Unclear | N/A | Low | High | **High** |
| Maniecka-Bryla, I. (2013) | Low | Low | Unclear | High | High | **High** |
| Martin-Fernandez, J. (2014) | Low | Low | Low | Low | High | **Low** |
| Melis, G., et al. (2015) | Low | Low | Unclear | Low | High | **High** |
| Mendes, L.L. (2013) | Low | Low | Low | High | High | **High** |
| Migliore, E. (2009) | Low | High | Low | High | High | **High** |
| Modig, L., et al. (2009) | Low | Unclear | Unclear | High | High | **High** |
| Modrek, S. (2011) | Low | Low | Unclear | Low | High | **High** |
| Morisco, F. (2017) | Low | Low | Low | Low | High | **Low** |
| Neuberger, M. (2013) | Low | Unclear | N/A | Low | High | **High** |
| Nolasco, A. (2015) | High | High | Unclear | Low | N/A | **High** |
| Nolasco, A. (2014) | High | High | Unclear | Low | N/A | **High** |
| Pasetto, R. (2013) | High | High | Unclear | Low | High | **High** |
| Patel, M.M. (2010) | Low | Low | Low | High | High | **High** |
| Pinto-Sarmento, T.C. (2016) | Low | Low | Unclear | Low | High | **High** |
| Piovesan, C., et al. (2010) | Low | Low | Low | Low | High | **Low** |
| Pizzo, G. (2010) | Low | Low | High | High | High | **High** |
| Ramsay, S.E. (2008) | Low | High | Low | High | High | **High** |
| Ribeiro, A.I. (2016) | Low | Unclear | Unclear | Low | N/A | **High** |
| Ribeiro Dos Santos, E. (2016) | Low | Low | Low | High | High | **High** |
| Ristovska, G. (2009) | Low | Unclear | Low | High | High | **High** |
| Rosicova, K. (2015) | Low | Low | Unclear | Low | High | **High** |
| Sanchez-Barriga, J.J. (2012) | High | High | Unclear | Low | N/A | **High** |
| Sanderson, M. (2015) | Low | Low | High | Unclear | Unclear | **High** |
| Santiago, B.M. (2013) | Low | Low | Low | High | Unclear | **High** |
| Santos, S.L. (2016) | Low | Low | N/A | Low | N/A | **Low** |
| Scazufca, M. (2010) | Low | Low | Low | Low | High | **Low** |
| Schulz, A.J. (2008) | Low | Low | Low | Low | N/A | **Low** |
| Smigielski, J. (2013) | Low | Low | Unclear | Unclear | Unclear | **High** |
| Steer, S. (2014) | Low | Unclear | Low | Low | High | **High** |
| Sulander, T. (2012) | Low | Low | High | High | High | **High** |
| Sun, G. (2017) | Low | Unclear | Unclear | Low | High | **High** |
| Thorn, L.K. (2011) | Low | Low | Unclear | Low | High | **High** |
| Trachtenberg, A.J. (2014) | Low | Low | Unclear | Low | High | **High** |
| Tucker-Seeley, R.D. (2013) | Low | Low | High | High | High | **High** |
| Unrath, M. (2014) | Low | Low | Low | Low | High | **Low** |
| Vandenheede, H. (2014) | Low | Low | Low | Low | N/A | **Low** |
| Walsh, D. (2010) | Low | High | Unclear | Low | N/A | **High** |
| Willers, S.M. (2016) | Low | Unclear | N/A | Low | High | **High** |
| Wong, C.M. (2008) | Low | Unclear | N/A | Low | High | **High** |
| Yang, B.Y. (2017) | Low | Low | Low | Low | High | **Low** |
